# Goal-directed haemodynamic therapy (GDHT) in surgical patients: systematic review and meta-analysis of the impact of GDHT on post-operative pulmonary complications

**DOI:** 10.1186/s13741-020-00161-5

**Published:** 2020-10-15

**Authors:** Ahilanandan Dushianthan, Martin Knight, Peter Russell, Michael PW Grocott

**Affiliations:** 1 General Intensive Care Unit, University Hospital Southampton NHS Foundation Trust, Southampton, SO16 6YD UK; 2 Anaesthesia Perioperative and Critical Care Research Group, Southampton NIHR Biomedical Research Centre, University Hospital Southampton/University of Southampton, Southampton, UK; 3 Clinical and Experimental Sciences, Faculty of Medicine, University of Southampton, Southampton, UK

**Keywords:** Goal-directed, Fluid therapy, Surgery

## Abstract

**Background:**

Perioperative goal-directed haemodynamic therapy (GDHT), defined as the administration of fluids with or without inotropes or vasoactive agents against explicit measured goals to augment blood flow, has been evaluated in many randomised controlled trials (RCTs) over the past four decades. Reported post-operative pulmonary complications commonly include chest infection or pneumonia, atelectasis, acute respiratory distress syndrome or acute lung injury, aspiration pneumonitis, pulmonary embolism, and pulmonary oedema. Despite the substantial clinical literature in this area, it remains unclear whether their incidence is reduced by GDHT. This systematic review aims to determine the effect of GDHT on the respiratory outcomes listed above, in surgical patients.

**Methods:**

We searched the Cochrane Central Register for Controlled Trials (CENTRAL), MEDLINE, EMBASE, and clinical trial registries up until January 2020. We included all RCTs reporting pulmonary outcomes. The primary outcome was post-operative pulmonary complications and secondary outcomes were specific pulmonary complications and intra-operative fluid input. Data synthesis was performed on Review Manager and heterogeneity was assessed using *I*^2^ statistics.

**Results:**

We identified 66 studies with 9548 participants reporting pulmonary complications. GDHT resulted in a significant reduction in total pulmonary complications (OR 0.74, 95% CI 0.59 to 0.92). The incidence of pulmonary infections, reported in 45 studies with 6969 participants, was significantly lower in the GDHT group (OR 0.72, CI 0.60 to 0.86). Pulmonary oedema was recorded in 23 studies with 3205 participants and was less common in the GDHT group (OR 0.47, CI 0.30 to 0.73). There were no differences in the incidences of pulmonary embolism or acute respiratory distress syndrome. Sub-group analyses demonstrated: (i) benefit from GDHT in general/abdominal/mixed and cardiothoracic surgery but not in orthopaedic or vascular surgery; and (ii) benefit from fluids with inotropes and/or vasopressors in combination but not from fluids alone. Overall, the GDHT group received more colloid (+280 ml) and less crystalloid (−375 ml) solutions than the control group. Due to clinical and statistical heterogeneity, we downgraded this evidence to moderate.

**Conclusions:**

This systematic review and meta-analysis suggests that the use of GDHT using fluids with inotropes and/or vasopressors, but not fluids alone, reduces the development of post-operative pulmonary infections and pulmonary oedema in general, abdominal and cardiothoracic surgical patients. This evidence was graded as moderate.

PROSPERO registry reference: CRD42020170361

## Introduction

Albeit uncommon, major surgery can be associated with significant pulmonary complications, increasing morbidity and mortality with a consequent greater burden on resources.(Pearse et al., 2012; Canet et al., 2010; Eappen et al., 2013) Goal-directed haemodynamic therapy (GDHT) describes the application of haemodynamic management to target-specific goals with the aim of improving global blood flow, and hence oxygen delivery, to the tissues. Previous systematic reviews support the notion that GDHT reduces post-operative complications following major surgery.(Hamilton et al., 2011) The role of GDHT in improving pulmonary outcomes following major surgery is uncertain.

The reported incidence of post-operative pulmonary complications varies depending on several factors including the definitions utilised, type of surgery, and patient-related variables including age, body mass index (BMI), functional reserve, smoking status, pre-existing lung diseases and the presence of obstructive sleep apnoea. The commonly reported pulmonary complications are atelectasis, respiratory tract infection/pneumonia, aspiration pneumonitis, acute respiratory distress syndrome (ARDS)/acute lung injury (ALI), pulmonary embolism and pulmonary oedema. Strategies to prevent or minimise post-operative pulmonary morbidity include pre-operative optimisation of pre-existing lung conditions, smoking cessation, pre-habilitation exercise programmes and intra-operative anaesthetic techniques such as increased use of neuraxial techniques, reduced use of long-acting neuromuscular blockade and lung-protective ventilation.(Ruscic et al., 2017)

In a Cochrane systematic review, the use of goal-directed fluid therapy was associated with a significant reduction in the incidence of respiratory failure.(Grocott et al., 2012) However, the effect of goal-directed fluid therapy on overall respiratory morbidity following surgery is unknown. Since the publication of the Cochrane review in 2012, many more randomised controlled trials have been published investigating the clinical impact of goal-directed haemodynamic therapy on post-operative outcomes, including respiratory complications.(Chong et al., 2018) The primary objective of this systematic review and meta-analysis is to investigate the effect of GDHT, defined by specific goals to augment blood flow, on reported post-operative pulmonary complications in patients undergoing surgery.

## Methods

We performed a systematic review and meta-analysis of all relevant published randomised controlled trials. We adhered to the Cochrane and PRISMA statement in reporting this systematic review and meta-analysis. We conducted a systematic search on Ovid MEDLINE (January 03, 2020), EMBASE (week 52 2019) and the Cochrane Central Register of Controlled Trials (January 03, 2020). The search strategies used are available in the supplementary material. The protocol for this study is registered and accessible on the PROSPERO (International Prospective Register of Systematic Reviews) Registry (CRD42020170361).

### Inclusion and exclusion criteria

We included all relevant randomised controlled trials with or without blinding and published in the English language. The participants were adults (age > 16 years) undergoing any elective or emergency surgery (abdominal, vascular, orthopaedic, cardiac, thoracic and mixed groups). Studies of non-surgical patients with trauma or sepsis were excluded. Peri-operative goal-directed haemodynamic therapy was defined as administration of fluids, with or without inotropes or vasoactive agents, against explicit measured goals to augment blood flow and initiated in the pre-operative (≤24 h), intra-operative or post-operative period (≤6 h). We included all invasive and non-invasive monitoring devices used with a pre-defined algorithm to augment flow towards specific haemodynamic targets including the following measured or derived variables: cardiac output (CO), stroke volume (SV), oxygen delivery (DO_2_), oxygen consumption (VO_2_), mixed venous oxygen saturation and oxygen extraction ratio (O_2_ER). We grouped haemodynamic monitoring techniques into 4 categories: pulmonary artery catheter, oesophageal Doppler, minimally invasive and non-invasive. We defined minimally invasive techniques as using devices that require insertion of an arterial line and/or central venous access (but did not require a pulmonary artery catheter) and used arterial pulse contour analysis, transpulmonary thermo- or lithium dilution to measure dynamic cardiac variables (calibrated or uncalibrated). More recently, studies have evaluated hemodynamic monitoring techniques with the use of bioimpedance/bioreactance and non-invasive pulse contour analysis. These devices were defined as non-invasive cardiac output monitoring. Studies based on a dynamic assessment of plasma volume and fluid responsiveness such as systolic pressure variation (SPV), pulse pressure variation (PPV) or plethysmography variability index (PVI) were excluded as they do not provide a measure of flow.

### Data collection

Two review authors (PR and MK) independently screened titles and abstracts from the search results to identify all potential studies. The studies were selected according to the inclusion and exclusion criteria and a third independent author (AD) further assessed studies against the eligibility criteria and resolved any disagreements by discussion. From the eligible studies, relevant data such as methods (study design, setting, number of centres), type of surgery and participants, interventions (goal-directed haemodynamic therapy algorithm and devices) and outcomes were extracted. Where data was missing, incomplete or grouped with other outcomes, we attempted to contact authors to obtain additional details if the study was within the 10-year period.

### Types of outcome measures

We included all studies that reported post-operative pulmonary complications. Our primary outcome was to assess the impact of goal-directed haemodynamic therapy on the incidence of post-operative pulmonary complications. This was presented as the total number of pulmonary events. We assessed individual events as secondary outcomes, as reported by the study authors. We used author-defined definitions for pneumonia, ARDS or ALI, pulmonary oedema, and pulmonary embolism. As it is often difficult clinically to distinguish pulmonary oedema associated with lung injury from fluid overload/hydrostatic or cardiogenic pulmonary oedema, we included this outcome as a pulmonary complication unless otherwise stated as cardiogenic in origin. We also performed subgroup analyses for the type of surgery, the haemodynamic monitoring device used, and the GDHT intervention delivered (fluids, or fluids and inotropes/vasopressors). We assessed intra-operative fluid administration, separating the use of crystalloids and colloids during the study period when reported.

### Assessment of risk of bias

We used the Cochrane risk of bias tool to assess the quality of included studies.(Higgins & Green, 2011) The risk of bias assessment consisted of the quality of randomisation and allocation concealment, blinding of participants and outcome assessors, selective or incomplete outcome reporting and any other potential sources of bias such as deviation from protocol, issues with the conduct of the study or influence from industry funding. We graded these components as “low risk”, “high risk” or “unclear risk”.

### Statistical analysis

Quantitative synthesis was performed on the RevMan 5.3 software (Review Manager 2014). To assess our dichotomous data, we used Odds Ratios (OR), using the Mantel-Haenszel method with random-effects and fixed-effects models with 95% confidence intervals (CI). Where possible, all our analysis was intention-to-treat (ITT). We assessed statistical heterogeneity by using *I*^2^ statistics.(Higgins & Green, 2011) Statistical heterogeneity was assumed when this value was >40% and we performed random-effects models for these analyses. When *I*^2^ was <40%, we used a fixed-effect model. Clinical heterogeneity was explored and addressed where possible by subgroup analysis. Consequently, we conducted subgroup analyses for the categories of surgery and the types of device utilised for the measurement of the specific haemodynamic goals. We encountered unit analysis issues, where some studies reported the number of patients with pulmonary complications, whilst others reported the total number of events within a population. Although we have attempted to analyse these separately, we did not proceed as there were only three studies that reported the number of patients with pulmonary complications. Data was transformed when no additional information was available to generate standard deviation and mean values from the median data presented (interquartile range, range, 95% confidence intervals, and 25th and 75th percentiles), as guided by the Cochrane Collaboration.(Higgins & Green, 2011) The quality of evidence was assessed by the Grading of Recommendations Assessment, Development and Evaluation (GRADE) system for the key outcomes reported.(Guyatt et al., 2008)

## Results

### Search results and study characteristics

The electronic search yielded a total of 18341 citations from electronic searches and additional records identified from other sources. After the removal of duplicates and screening by title and abstracts, we retrieved 150 studies for detailed review. We further excluded abstract publications (*N* = 30), dynamic studies which track pulse pressure or systolic pressure variations with no surrogate indices for global blood flow (*N* = 13) and studies that used additional goal-directed measures in the control group or compared different fluid solutions in the GDHT setting (*N* = 11). Ninety-six randomised controlled trials fulfilled our inclusion criteria and of those 67 studies reported post-operative pulmonary complications. One study reported pooled cardiac and respiratory complications and as a result, this was not included for quantitative data synthesis.(Srinivasa et al., 2013) We identified 6 ongoing trials (Table 1).

**Table 1:** Ongoing studies identified from trial registries

Title	Registry Number	Expected completion
A clinical trial of blood flow optimisation for patients who have emergency bowel surgery (FLOELA)	ISRCTN14729158	January 2022
Optimisation of perioperative cardiovascular management to improve surgical outcome II (OPTIMISE II)	ISRCTN39653756	September 2021
Effect of SVV-guided fluid therapy on outcomes after major abdominal surgery	NCT03940144	August 2020
Management of intraoperative fluids in ambulatory surgery (MIFAS)	NCT03193320	December 2020
Effects of goal-directed fluid therapy on postoperative outcomes in patients undergoing laparoscopic colorectal surgery in different levels of systemic vascular resistance after general anaesthesia	ChiCTR1900022775	Ongoing
The Application of Goal-directed fluid therapy in the Fast-track Anaesthesia for Gastrectomy	ChiCTR-INR-17010636	Ongoing

**Fig. 1:**
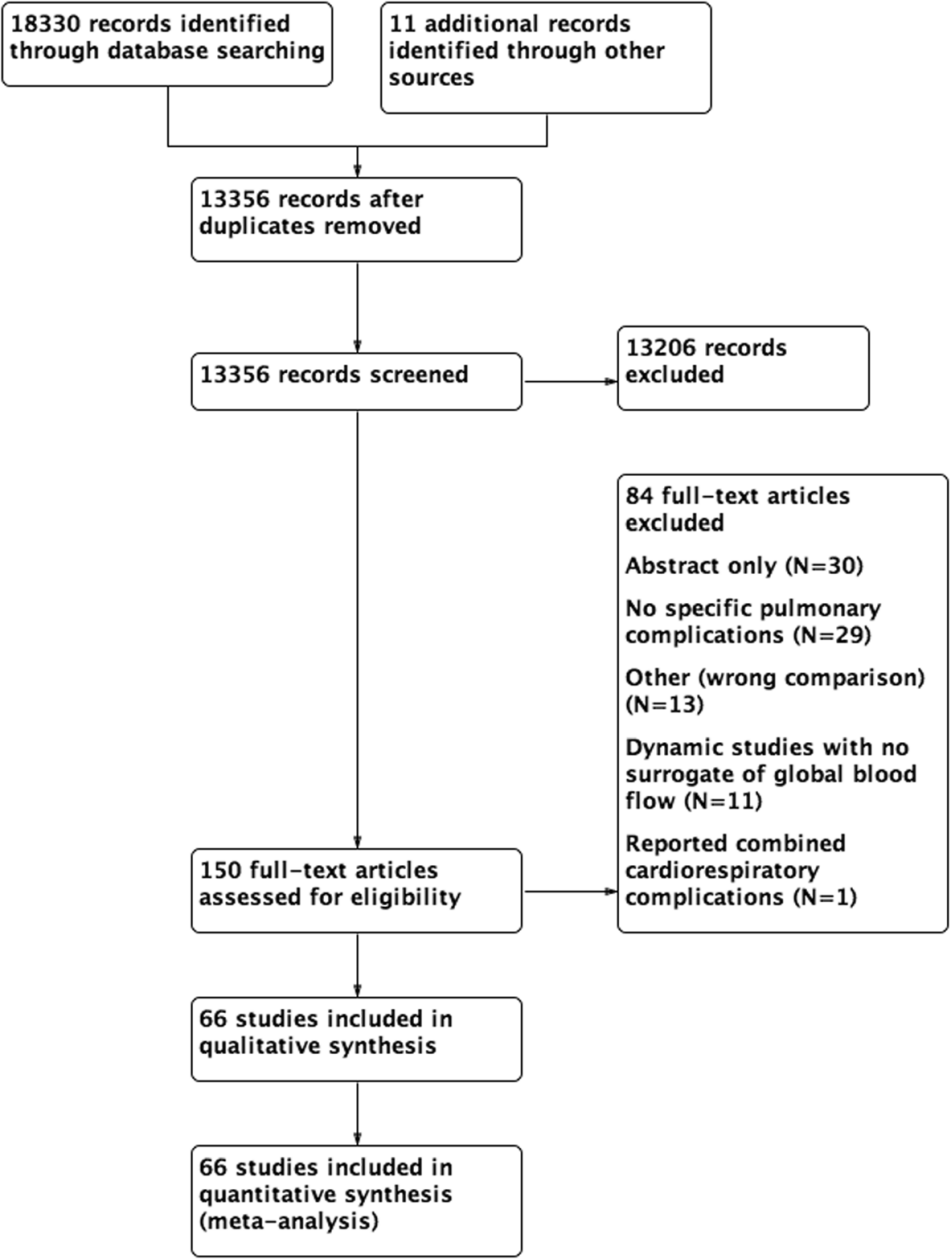
PRISMA study search flow diagram

We also screened references from all eligible publications and other systematic reviews for additional publications. The PRISMA flow chart for the study search flow diagram is presented in Fig. 1.

A total of 66 randomised controlled trials with 9548 participants reported respiratory complications as part of their outcome reporting. A summary table of study characteristics is presented in Table 2, and an additional detailed table is available in the Supplementary material (Table A). The type of surgery performed was general/mixed group or abdominal (44 studies)(Ackland et al., 2015; Bahlmann et al., 2019; Benes et al., 2010; Boyd et al., 1993; Brandstrup et al., 2012; Calvo-Vecino et al., 2018; Colantonio et al., 2015; Correa-Gallego et al., 2015; Donati et al., 2007; El Sharkawy et al., 2013; Gan et al., 2002; Gerent et al., 2018; Gomez-Izquierdo et al., 2017; Jammer et al., 2010; Jammer et al., 2015; Joosten et al., 2019; Kim et al., 2018; Kumar et al., 2016; Lobo et al., 2000; Luo et al., 2017; Mayer et al., 2010; McKenny et al., 2013; Mikor et al., 2015; Pearse et al., 2005; Pearse et al., 2014; Pestana et al., 2014; Phan et al., 2014; Reisinger et al., 2017; Salzwedel et al., 2013; Sandham et al., 2003; Scheeren et al., 2013; Schmid et al., 2016; Senagore et al., 2009; Shoemaker et al., 1988; Stens et al., 2017; Ueno et al., 1998; Wakeling et al., 2005; Weinberg et al., 2017; Weinberg et al., 2019; Wilson et al., 1999; Wu et al., 2017; Yin et al., 2018; Zakhaleva et al., 2013; Zeng et al., 2014), orthopaedic (9 studies)(Bartha et al., 2013; Cecconi et al., 2011; Davies et al., 2019; Han et al., 2016; Kaufmann et al., 2018; Moppett et al., 2015; Peng et al., 2014; Sinclair et al., 1997; Venn et al., 2002), cardiothoracic (7 studies) (Goepfert et al., 2013; Kaufmann et al., 2017; McKendry et al., 2004; Mythen & Webb, 1995; Osawa et al., 2016; Xu et al., 2017; Zhang et al., 2013), and vascular (6 studies)(Bender et al., 1997; Bisgaard et al., 2013b; Boyd et al., 1993; Funk et al., 2015; Valentine et al., 1998; Van der Linden et al., 2010). Five studies were solely conducted on orthopaedic emergency patients(Bartha et al., 2013; Davies et al., 2019; Moppett et al., 2015; Sinclair et al., 1997; Venn et al., 2002), six additional studies included emergency surgical patients(Boyd et al., 1993; McKendry et al., 2004; Pearse et al., 2005; Pearse et al., 2014; Sandham et al., 2003; Shoemaker et al., 1988) and the rest were primarily conducted on elective surgical patients. The total sample size was <100 participants for 33 studies(Bahlmann et al., 2019; Bisgaard et al., 2013a; Bisgaard et al., 2013b; Cecconi et al., 2011; Colantonio et al., 2015; El Sharkawy et al., 2013; Funk et al., 2015; Han et al., 2016; Jammer et al., 2015; Joosten et al., 2019; Kaufmann et al., 2018; Kim et al., 2018; Kumar et al., 2016; Lobo et al., 2000; Mayer et al., 2010; Mikor et al., 2015; Mythen & Webb, 1995; Peng et al., 2014; Reisinger et al., 2017; Scheeren et al., 2013; Senagore et al., 2009; Shoemaker et al., 1988; Sinclair et al., 1997; Ueno et al., 1998; Van der Linden et al., 2010; Venn et al., 2002; Weinberg et al., 2017; Weinberg et al., 2019; Wu et al., 2017; Yin et al., 2018; Zakhaleva et al., 2013; Zeng et al., 2014; Zhang et al., 2013) (50%) and the majority (77%) were conducted in a single centre setting(Bartha et al., 2013; Bender et al., 1997; Benes et al., 2010; Bisgaard et al., 2013a; Bisgaard et al., 2013b; Boyd et al., 1993; Cecconi et al., 2011; Colantonio et al., 2015; Correa-Gallego et al., 2015; El Sharkawy et al., 2013; Funk et al., 2015; Gan et al., 2002; Gerent et al., 2018; Goepfert et al., 2013; Gomez-Izquierdo et al., 2017; Han et al., 2016; Joosten et al., 2019; Kaufmann et al., 2017; Kaufmann et al., 2018; Kim et al., 2018; Kumar et al., 2016; Lobo et al., 2000; Luo et al., 2017; Mayer et al., 2010; McKendry et al., 2004; McKenny et al., 2013; Mikor et al., 2015; Moppett et al., 2015; Mythen & Webb, 1995; Osawa et al., 2016; Pearse et al., 2005; Peng et al., 2014; Phan et al., 2014; Reisinger et al., 2017; Salzwedel et al., 2013; Schmid et al., 2016; Senagore et al., 2009; Shoemaker et al., 1988; Sinclair et al., 1997; Ueno et al., 1998; Valentine et al., 1998; Van der Linden et al., 2010; Venn et al., 2002; Wakeling et al., 2005; Wilson et al., 1999; Wu et al., 2017; Xu et al., 2017; Yin et al., 2018; Zakhaleva et al., 2013; Zeng et al., 2014; Zhang et al., 2013). The monitoring device for the majority of the studies was minimally invasive (33 studies)(Ackland et al., 2015; Bahlmann et al., 2019; Bartha et al., 2013; Benes et al., 2010; Bisgaard et al., 2013a; Bisgaard et al., 2013b; Cecconi et al., 2011; Colantonio et al., 2015; Correa-Gallego et al., 2015; Funk et al., 2015; Gerent et al., 2018; Goepfert et al., 2013; Han et al., 2016; Jammer et al., 2015; Kim et al., 2018; Kumar et al., 2016; Luo et al., 2017; Mayer et al., 2010; Moppett et al., 2015; Osawa et al., 2016; Pearse et al., 2005; Pearse et al., 2014; Peng et al., 2014; Salzwedel et al., 2013; Scheeren et al., 2013; Schmid et al., 2016; Van der Linden et al., 2010; Weinberg et al., 2017; Weinberg et al., 2019; Wu et al., 2017; Xu et al., 2017; Zeng et al., 2014; Zhang et al., 2013), followed by oesophageal Doppler (17 studies)(Brandstrup et al., 2012; Calvo-Vecino et al., 2018; El Sharkawy et al., 2013; Gan et al., 2002; Gomez-Izquierdo et al., 2017; Kaufmann et al., 2017; Kaufmann et al., 2018; McKendry et al., 2004; McKenny et al., 2013; Mythen & Webb, 1995; Phan et al., 2014; Reisinger et al., 2017; Senagore et al., 2009; Sinclair et al., 1997; Venn et al., 2002; Wakeling et al., 2005; Zakhaleva et al., 2013), pulmonary artery catheter (8 studies)(Bender et al., 1997; Boyd et al., 1993; Lobo et al., 2000; Sandham et al., 2003; Shoemaker et al., 1988; Ueno et al., 1998; Valentine et al., 1998; Wilson et al., 1999), non-invasive cardiac output monitor (5 studies)(Davies et al., 2019; Joosten et al., 2019; Pestana et al., 2014; Stens et al., 2017; Yin et al., 2018) and those measuring oxygen parameters (3 studies)(Donati et al., 2007; Jammer et al., 2010; Mikor et al., 2015). Goal-directed haemodynamic therapy was initiated intra-operatively in the majority of studies (82%)(Bahlmann et al., 2019; Benes et al., 2010; Bisgaard et al., 2013a; Bisgaard et al., 2013b; Brandstrup et al., 2012; Calvo-Vecino et al., 2018; Cecconi et al., 2011; Colantonio et al., 2015; Correa-Gallego et al., 2015; Davies et al., 2019; Donati et al., 2007; El Sharkawy et al., 2013; Funk et al., 2015; Gan et al., 2002; Goepfert et al., 2013; Gomez-Izquierdo et al., 2017; Han et al., 2016; Jammer et al., 2010; Jammer et al., 2015; Joosten et al., 2019; Kaufmann et al., 2017; Kaufmann et al., 2018; Kim et al., 2018; Kumar et al., 2016; Lobo et al., 2000; Luo et al., 2017; Mayer et al., 2010; McKenny et al., 2013; Mikor et al., 2015; Moppett et al., 2015; Mythen & Webb, 1995; Osawa et al., 2016; Pearse et al., 2014; Peng et al., 2014; Pestana et al., 2014; Phan et al., 2014; Reisinger et al., 2017; Salzwedel et al., 2013; Scheeren et al., 2013; Schmid et al., 2016; Senagore et al., 2009; Sinclair et al., 1997; Stens et al., 2017; Van der Linden et al., 2010; Venn et al., 2002; Wakeling et al., 2005; Weinberg et al., 2017; Weinberg et al., 2019; Wu et al., 2017; Xu et al., 2017; Yin et al., 2018; Zakhaleva et al., 2013; Zeng et al., 2014; Zhang et al., 2013). In 22 studies (33%), the GDHT intervention used fluids alone(Brandstrup et al., 2012; Correa-Gallego et al., 2015; El Sharkawy et al., 2013; Gan et al., 2002; Gomez-Izquierdo et al., 2017; Han et al., 2016; Jammer et al., 2010; Joosten et al., 2019; Kim et al., 2018; McKenny et al., 2013; Moppett et al., 2015; Mythen & Webb, 1995; Peng et al., 2014; Phan et al., 2014; Reisinger et al., 2017; Scheeren et al., 2013; Senagore et al., 2009; Sinclair et al., 1997; Venn et al., 2002; Wakeling et al., 2005; Zakhaleva et al., 2013; Zeng et al., 2014) and the rest used fluids with vasoactive or inotropic agents as part of their GDHT protocol(Ackland et al., 2015; Bahlmann et al., 2019; Bartha et al., 2013; Bender et al., 1997; Benes et al., 2010; Bisgaard et al., 2013a; Bisgaard et al., 2013b; Boyd et al., 1993; Calvo-Vecino et al., 2018; Cecconi et al., 2011; Colantonio et al., 2015; Davies et al., 2019; Donati et al., 2007; Funk et al., 2015; Gerent et al., 2018; Goepfert et al., 2013; Jammer et al., 2015; Kaufmann et al., 2017; Kaufmann et al., 2018; Kim et al., 2018; Lobo et al., 2000; Luo et al., 2017; Mayer et al., 2010; McKendry et al., 2004; Mikor et al., 2015; Osawa et al., 2016; Pearse et al., 2005; Pearse et al., 2014; Pestana et al., 2014; Salzwedel et al., 2013; Sandham et al., 2003; Schmid et al., 2016; Shoemaker et al., 1988; Stens et al., 2017; Ueno et al., 1998; Valentine et al., 1998; Van der Linden et al., 2010; Weinberg et al., 2017; Weinberg et al., 2019; Wilson et al., 1999; Wu et al., 2017; Xu et al., 2017; Yin et al., 2018; Zhang et al., 2013). Among those which used inotropes, more than 50% used dobutamine(Ackland et al., 2015; Bahlmann et al., 2019; Bartha et al., 2013; Benes et al., 2010; Bisgaard et al., 2013a; Bisgaard et al., 2013b; Cecconi et al., 2011; Donati et al., 2007; Gerent et al., 2018; Kim et al., 2018; Kumar et al., 2016; Lobo et al., 2000; Mayer et al., 2010; Osawa et al., 2016; Pestana et al., 2014; Salzwedel et al., 2013; Schmid et al., 2016; Shoemaker et al., 1988; Stens et al., 2017; Ueno et al., 1998; Van der Linden et al., 2010; Wu et al., 2017; Xu et al., 2017; Yin et al., 2018; Zhang et al., 2013). The fluid bolus in the GDHT algorithm consisted of a colloid solution in nearly two-thirds of the studies (Cecconi et al., 2011; Chappell et al., 2008; Chong et al., 2018; Correa-Gallego et al., 2015; Davies et al., 2019; Donati et al., 2007; Eappen et al., 2013; El Sharkawy et al., 2013; Evans et al., 2009; Funk et al., 2015; Gerent et al., 2018; Goepfert et al., 2013; Gomez-Izquierdo et al., 2017; Grocott et al., 2012; Guyatt et al., 2008; Hamilton et al., 2011; Han et al., 2016; Higgins & Green, 2011; Joosten et al., 2019; Kim et al., 2018; Lobo et al., 2000; Luo et al., 2017; Mayer et al., 2010; McKendry et al., 2004; McKenny et al., 2013; Moppett et al., 2015; Mythen & Webb, 1995; Noblett et al., 2006; Osawa et al., 2016; Pearse et al., 2005; Pearse et al., 2014; Pestana et al., 2014; Phan et al., 2014; Ruscic et al., 2017; Schmid et al., 2016; Senagore et al., 2009; Shoemaker et al., 1988; Ueno et al., 1998; Valentine et al., 1998; Van der Linden et al., 2010; Wakeling et al., 2005; Weinberg et al., 2017). Nine studies used a combination of crystalloids and colloids(Calvo-Vecino et al., 2018; Gerent et al., 2018; Kumar et al., 2016; Lobo et al., 2000; Mayer et al., 2010; Senagore et al., 2009; Shoemaker et al., 1988; Stens et al., 2017; Wilson et al., 1999) and a further 9 studies used crystalloids alone for fluid boluses as part of their GDHT protocol (Colantonio et al., 2015; Gan et al., 2002; http://www.isrctn.com/ISRCTN14729158, n.d.; Jammer et al., 2015; Jammer et al., 2010; Jhanji et al., 2010; Mikor et al., 2015; Scheeren et al., 2013; Sinclair et al., 1997).

**Table 2:** Summary characteristics of included studies.

Author/year	No. of patients	Single/multicenter	Type of surgery	Timing	Fluids/fluids & inotropes (I) or vasopressors (V)	Goals
Ackland, 2015	204	Multi	Elective	Post-operative	Fluids & I/V	SV, CO, DO2I
Bahlmann, 2019	59	Multi	Elective	Intra/post-operative	Fluids & I/V	SV, CI, MAP
Bartha, 2013	149	Single	Emergency	Pre/intra-operative	Fluids & I/V	SV, DO_2_I
Bender 1997	104	Single	Elective	Pre/intra/post-operative	Fluids & I/V	SVR, CI, PAOP
Benes, 2010	120	Single	Elective	Intra-operative	Fluids & I/V	SVV, CI
Bisgaard, 2013a, b	70	Single	Elective	Intra/post-operative	Fluids & I/V	SVI, DO_2_I
Bisgaard, 2013a, b	40	Single	Elective	Intra/post-operative	Fluids & I/V	SVI, DO_2_I
Boyd, 1993	107	Single	Mixed	Pre/intra/post-operative	Fluids & I/V	DO_2_I
Brandstrup, 2012	151	Multi	Elective	Intra-operative	Fluids	SV
Calvo-Vecino, 2018	450	Multi	Elective	Intra-operative	Fluids & I/V	SV, CI
Cecconi, 2011	40	Single	Elective	Intra/post-operative	Fluids & I/V	SV, DO_2_I
Colantonio, 2015	86	Single	Elective	Intra-operative	Fluids & I/V	SVI, CI
Correa-Gallego, 2015	135	Single	Elective	Intra-operative	Fluids	SVV
Davies, 2019	241	Multi	Emergency	Intra-operative	Fluids	SV
Donati, 2007	135	Multi	Elective	Intra/post-operative	Fluids & I/V	O_2_ER
El-Sharkawy, 2013	59	Single	Elective	Intra/post-operative	Fluids	SV, FTc
Funk, 2015	40	Single	Elective	Intra-operative	Fluids & I/V	SVV, CI
Gan, 2002	100	Single	Elective	Intra-operative	Fluids	SV, FTc
Gerent, 2018	128	Single	Elective	Post-operative	Fluids & I/V	SVI, CI
Goepfert, 2013	100	Single	Elective	Intra/post-operative	Fluids & I/V	GEDI, SVV, CI
Gómez-Izquierdo, 2017	135	Single	Elective	Intra-operative	Fluids	SV
Han, 2016	40	Single	Elective	Intra-operative	Fluids	SVV
Jammer, 2010	241	Multi	Elective	Intra/post-operative	Fluids	ScVO_2_
Jammer, 2015	30	Multi	Elective	Intra-operative	Fluids & I/V	SVV
Joosten, 2019	39	Single	Elective	Intra-operative	Fluids	CI, SVV
Kaufmann, 2017	100	Single	Elective	Intra-operative	Fluids & I/V	SV, CI
Kaufmann, 2018	90	Single	Mixed	Intra-operative	Fluids & I/V	SV, CI
Kim, 2018	62	Single	Elective	Intra-operative	Fluids & I/V	SVV, CI
Kumar, 2016	60	Single	Elective	Intra-operative	Fluids & I/V	SVV, SVR
Lobo, 2000	37	Single	Elective	Intra/post-operative	Fluids & I/V	DO_2_, PAOP
Luo, 2017	150	Single	Elective	Intra-operative	Fluids & I/V	SVV, CI
Mayer, 2010	60	Single	Elective	Intra-operative	Fluids & I/V	CI, SVI
McKendry, 2004	179	Single	Mixed	Post-operative	Fluids & I/V	SI
McKenny, 2013	102	Single	Elective	Intra-operative	Fluids	SV
Mikor, 2015	84	Single	Elective	Intra-operative	Fluids & I/V	ScVO_2_
Moppett, 2015	130	Single	Emergency	Intra-operative	Fluids	SV
Mythen, 1995	60	Single	Elective	Intra-operative	Fluids	SV, CVP
Osawa, 2016	126	Single	Elective	Intra/post-operative	Fluids & I/V	SVI, CI
Pearse, 2005	122	Single	Mixed	Post-operative	Fluids & I/V	SV, DO_2_I, CI
Pearse, 2014	734	Multi	Mixed	Intra/post-operative	Fluids & I/V	SV
Peng, 2014	80	Single	Elective	Intra-operative	Fluids	SVV
Pestaña, 2014	170	Multi	Elective	Intra/post-operative	Fluids & I/V	SV, CI, MAP
Phan, 2014	100	Single	Elective	Intra-operative	Fluids	SVI, FTc
Reisinger, 2017	58	Single	Elective	Intra/post-operative	Fluids	SVI
Salzwedel, 2013	180	Multi	Elective	Intra-operative	Fluids & I/V	PPV, CI
Sandham, 2003	1994	Multi	Mixed	Pre-operative	Fluids & I/V	DO_2_I, CI
Scheeren, 2013	64	Multi	Elective	Intra-operative	Fluids	SVV, SV
Schmid, 2016	193	Single	Elective	Intra/post-operative	Fluids & I/V	GEDI, CI
Senagore, 2009	64	Single	Elective	Intra-operative	Fluids	SV
Shoemaker, 1998	88	Single	Mixed	Pre-operative	Fluids & I/V	CI, DO_2_I, VO_2_I
Sinclair, 1997	40	Single	Emergency	Intra-operative	Fluids	SV, FTc
Stens, 2017	244	Multi	Elective	Intra-operative	Fluids & I/V	CI, PPV
Ueno, 1998	34	Single	Elective	Post-operative	Fluids & I/V	CI, DO_2_I, VO2I
Valentine, 1998	120	Single	Elective	Pre-operative	Fluids & I/V	PCWP, CI, SVR
Van der Linden, 2010	57	Single	Elective	Intra-operative	Fluids & I/V	CI
Venn, 2002	90	Single	Emergency	Intra-operative	Fluids	SV, FTc
Wakeling, 2005	134	Single	Elective	Intra-operative	Fluids	SV
Weinberg, 2017	52	Multi	Elective	Intra-operative	Fluids & I/V	SVV, CI
Weinberg, 2019	50	Multi	Elective	Intra-operative	Fluids & I/V	SVV, CI
Wilson, 1999	138	Single	Elective	Pre/intra/post-operative	Fluids & I/V	DO_2_I
Wu, 2017	66	Single	Elective	Intra-operative	Fluids & I/V	SVV, CI
Xu, 2017	172	Single	Elective	Intra-operative	Fluids & I/V	SVV, CI
Yin, 2018	50	Single	Elective	Intra-operative	Fluids & I/V	SVV, CI
Zakhaleva 2013	91	Single	Elective	Intra-operative	Fluids	SV, FTc
Zeng, 2014	60	Single	Elective	Intra-operative	Fluids	SVV
Zhang, 2013	60	Single	Elective	Intra-operative	Fluids & I/V	SVV, CI

**Fig. 2:**
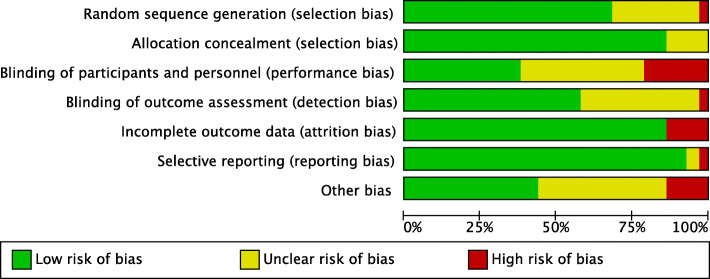
Risk of bias graph: review authors’ judgements about each risk of bias item presented as percentages across all included studies. Green indicates no risk of bias, yellow and red represent unclear risk and high risk respectively.

All included studies were assessed for the risk of bias using the Cochrane Collaboration tool for assessing risk of bias.(Higgins & Green, 2011) Each study was assessed according to seven domains: random sequence generation, allocation concealment, blinding of participants and personnel, blinding of outcome assessment, incomplete outcome data, selective reporting and other biases. Random sequence generation was identified as at low risk of bias in 45 studies (68%) (Cecconi et al., 2011; Chappell et al., 2008; Chong et al., 2018; Correa-Gallego et al., 2015; Davies et al., 2019; Donati et al., 2007; Eappen et al., 2013; Edwards et al., 2019; Evans et al., 2009; Grocott et al., 2012; Grocott et al., 2005; Guyatt et al., 2008; Hamilton et al., 2011; Higgins & Green, 2011; http://www.isrctn.com/ISRCTN14729158, n.d.; Jammer et al., 2015; Jammer et al., 2010; Jhanji et al., 2010; Joosten et al., 2019; Kim et al., 2018; Lobo et al., 2000; McKendry et al., 2004; Mikor et al., 2015; Moppett et al., 2015; Mythen & Webb, 1995; Noblett et al., 2006; Osawa et al., 2016; Pearse et al., 2005; Pearse et al., 2014; Peng et al., 2014; Phan et al., 2014; Polonen et al., 2000; Reisinger et al., 2017; Sandham et al., 2003; Schmid et al., 2016; Senagore et al., 2009; Sinclair et al., 1997; Srinivasa et al., 2013; Stens et al., 2017; Valentine et al., 1998; Van der Linden et al., 2010; Venn et al., 2002). Allocation concealment was judged to be adequate in 57 studies (88%)(Ackland et al., 2015; Bahlmann et al., 2019; Bartha et al., 2013; Benes et al., 2010; Brandstrup et al., 2012; Calvo-Vecino et al., 2018; Cecconi et al., 2011; Colantonio et al., 2015; Correa-Gallego et al., 2015; Davies et al., 2019; El Sharkawy et al., 2013; Funk et al., 2015; Gan et al., 2002; Gerent et al., 2018; Goepfert et al., 2013; Gomez-Izquierdo et al., 2017; Jammer et al., 2010; Jammer et al., 2015; Joosten et al., 2019; Kaufmann et al., 2017; Kaufmann et al., 2018; Kumar et al., 2016; Lobo et al., 2000; Luo et al., 2017; Mayer et al., 2010; McKendry et al., 2004; McKenny et al., 2013; Mikor et al., 2015; Moppett et al., 2015; Mythen & Webb, 1995; Osawa et al., 2016; Pearse et al., 2005; Pearse et al., 2014; Peng et al., 2014; Pestana et al., 2014; Phan et al., 2014; Reisinger et al., 2017; Salzwedel et al., 2013; Sandham et al., 2003; Scheeren et al., 2013; Schmid et al., 2016; Senagore et al., 2009; Shoemaker et al., 1988; Sinclair et al., 1997; Stens et al., 2017; Valentine et al., 1998; Van der Linden et al., 2010; Venn et al., 2002; Wakeling et al., 2005; Weinberg et al., 2017; Weinberg et al., 2019; Wilson et al., 1999; Wu et al., 2017; Xu et al., 2017; Yin et al., 2018; Zakhaleva et al., 2013; Zhang et al., 2013). Blinding of participants and personnel was achieved adequately in only 25 studies (38%) (Cecconi et al., 2011; Correa-Gallego et al., 2015; Eappen et al., 2013; Edwards et al., 2019; El Sharkawy et al., 2013; Gan et al., 2002; Hamilton et al., 2011; Jammer et al., 2010; Jhanji et al., 2010; Lobo et al., 2000; McKendry et al., 2004; Moppett et al., 2015; Noblett et al., 2006; Osawa et al., 2016; Pearse et al., 2005; Pearse et al., 2014; Polonen et al., 2000; Ruscic et al., 2017; Schmid et al., 2016; Senagore et al., 2009; Shoemaker et al., 1988; Srinivasa et al., 2013; Ueno et al., 1998; Valentine et al., 1998; Van der Linden et al., 2010), and blinding of outcome in 38 (58%)(Ackland et al., 2015; Benes et al., 2010; Bisgaard et al., 2013a; Bisgaard et al., 2013b; Brandstrup et al., 2012; Calvo-Vecino et al., 2018; Colantonio et al., 2015; Correa-Gallego et al., 2015; Davies et al., 2019; Funk et al., 2015; Gan et al., 2002; Gerent et al., 2018; Goepfert et al., 2013; Gomez-Izquierdo et al., 2017; Jammer et al., 2010; Jammer et al., 2015; Joosten et al., 2019; Kaufmann et al., 2017; Kaufmann et al., 2018; Kim et al., 2018; Mayer et al., 2010; McKendry et al., 2004; McKenny et al., 2013; Moppett et al., 2015; Osawa et al., 2016; Pearse et al., 2005; Pearse et al., 2014; Peng et al., 2014; Pestana et al., 2014; Phan et al., 2014; Reisinger et al., 2017; Sandham et al., 2003; Scheeren et al., 2013; Van der Linden et al., 2010; Weinberg et al., 2017; Wu et al., 2017; Xu et al., 2017; Zeng et al., 2014). In nine studies (14%), there was incomplete outcome data(El Sharkawy et al., 2013; Jammer et al., 2015; Pestana et al., 2014; Salzwedel et al., 2013; Scheeren et al., 2013; Stens et al., 2017; Zakhaleva et al., 2013; Zeng et al., 2014; Zhang et al., 2013). Sixty-one studies (92%) were identified as at low risk of selective reporting bias(Ackland et al., 2015; Bahlmann et al., 2019; Bartha et al., 2013; Bender et al., 1997; Benes et al., 2010; Bisgaard et al., 2013a; Bisgaard et al., 2013b; Boyd et al., 1993; Brandstrup et al., 2012; Calvo-Vecino et al., 2018; Cecconi et al., 2011; Colantonio et al., 2015; Correa-Gallego et al., 2015; Davies et al., 2019; Donati et al., 2007; Funk et al., 2015; Gan et al., 2002; Gerent et al., 2018; Goepfert et al., 2013; Gomez-Izquierdo et al., 2017; Jammer et al., 2010; Joosten et al., 2019; Kaufmann et al., 2017; Kaufmann et al., 2018; Kim et al., 2018; Kumar et al., 2016; Luo et al., 2017; Mayer et al., 2010; McKendry et al., 2004; McKenny et al., 2013; Mikor et al., 2015; Moppett et al., 2015; Mythen & Webb, 1995; Osawa et al., 2016; Pearse et al., 2005; Pearse et al., 2014; Peng et al., 2014; Pestana et al., 2014; Phan et al., 2014; Reisinger et al., 2017; Salzwedel et al., 2013; Sandham et al., 2003; Scheeren et al., 2013; Schmid et al., 2016; Senagore et al., 2009; Shoemaker et al., 1988; Sinclair et al., 1997; Stens et al., 2017; Valentine et al., 1998; Van der Linden et al., 2010; Venn et al., 2002; Wakeling et al., 2005; Weinberg et al., 2017; Weinberg et al., 2019; Wilson et al., 1999; Wu et al., 2017; Xu et al., 2017; Yin et al., 2018; Zakhaleva et al., 2013; Zeng et al., 2014; Zhang et al., 2013). Multiple studies reported industry funding, which was considered a potential source of bias. In one study, the second author has been investigated for scientific misconduct(Mayer et al., 2010). The overall risk of bias is presented in Fig. 2 and a table of individual study bias is available in the Supplementary material (Figure A).

### Synthesis of results

#### Primary outcome

**Fig. 3:**
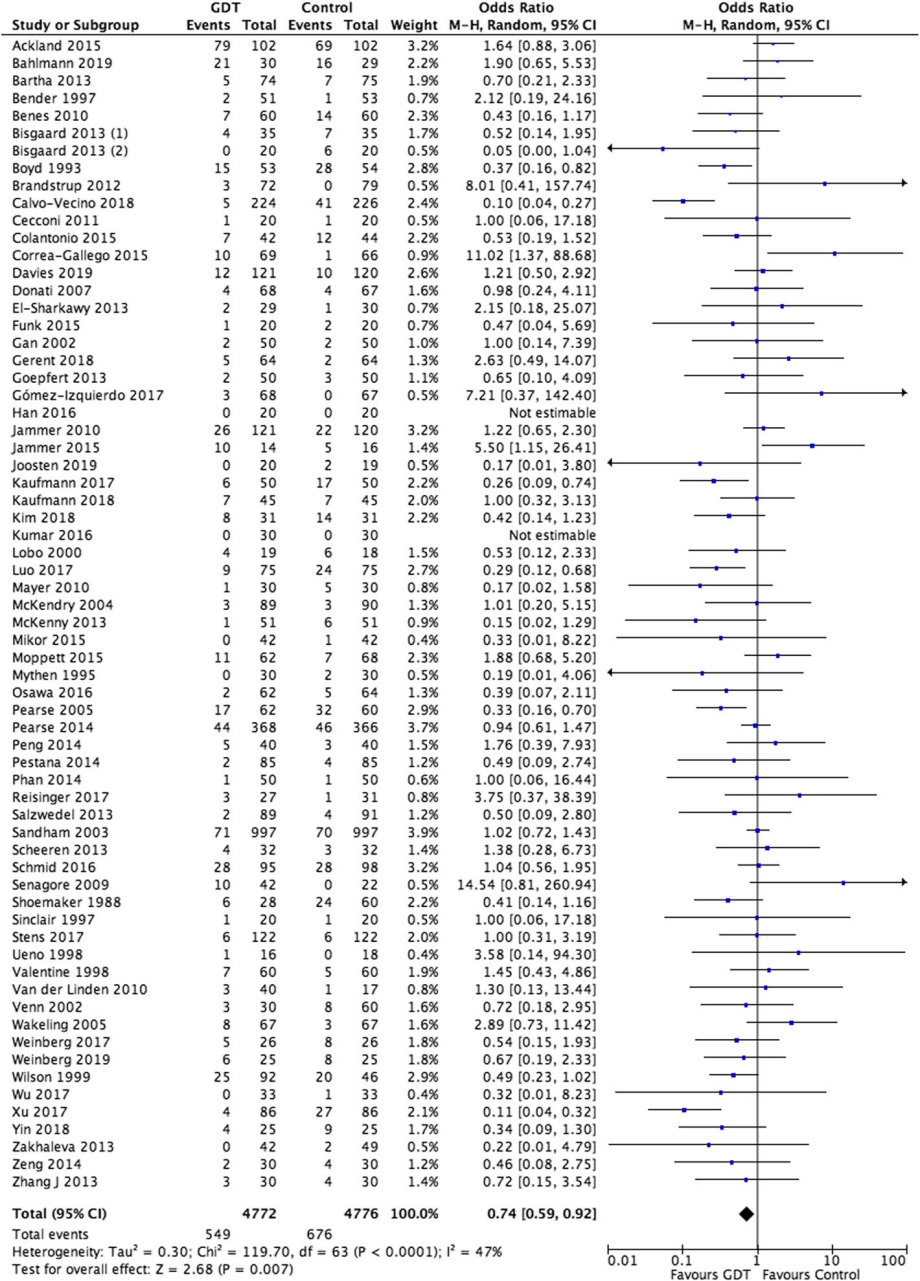
Forest plot for the primary outcome

**Fig. 4:**
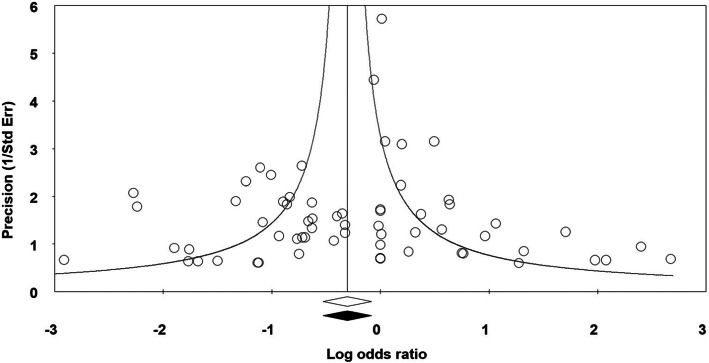
Funnel plot of precision by log odds ratio

Sixty-six studies with a total of 9548 participants reported post-operative pulmonary events. These respiratory events accounted for 11.5% (549/4772) of the GDHT group and 14% (676/4776) of the control group. There was a significant difference between groups (OR 0.74, 95% CI 0.59 to 0.92; *I*^2^ = 47%, *P* = 0.007) (Fig. 3). A random-effects model was utilised as there was moderate statistical heterogeneity. There was substantial clinical heterogeneity among the included studies with variations in age of study (1988 to 2019), study population, methodology, operative and GDHT interventions and measured outcomes. The quality of evidence was downgraded to moderate due to statistical and clinical heterogeneity. Funnel plot analysis suggested visually no significant asymmetry with Egger’s regression intercept of −0.1046 (95% CI −0.7659 to 0.5567) and *P* = 0.7530 (Fig. 4).

#### Secondary outcomes

We assessed individual respiratory outcomes such as pulmonary infections, ARDS, ALI, pulmonary embolism, and pulmonary oedema as secondary outcomes. Goal-directed haemodynamic therapy significantly reduced the incidence of post-operative respiratory tract infections or pneumonia (OR 0.72 [95% 0.60 to 0.86], *I*^2^ = 0%, *P* = 0.0003, 45 studies) and pulmonary oedema (OR 0.47 [ 95% CI 0.30 to 0.73], *I*^2^ = 0%, *P* = 0.0008, 23 studies). However, there were no differences in ARDS (OR 0.57 [95% CI 0.31 to 1.02], *I*^2^ = 17%, *P* = 0.06, 15 studies) or pulmonary embolism (OR 1.08 [95% CI 0.59 to 1.95], *I*^2^ = 1%, *P* = 0.81, 28 studies) (Table 3).

**Table 3:** Secondary outcome for specific pulmonary complications. *ARDS* acute respiratory distress syndrome, *CI* confidence interval, *M-H* Mantel-Haenszel

Outcome	No. of studies	No. of patients	Analysis	Effect (95% CI)	***P*** value
Chest infection or pneumonia	45	6969	M-H odds ratio	0.72 (0.60–0.86)	P = 0.0003
ARDS	15	2491	M-H odds ratio	0.57 (0.31–1.02)	P = 0.06
Pulmonary oedema	23	3205	M-H odds ratio	0.47 (0.30–0.73)	P = 0.0008
Pulmonary embolism	28	5430	M-H odds ratio	1.08 (0.59–1.95)	P = 0.81

The intra-operative fluid input and balance was presented in a number of different ways by the included studies. Overall, sixty-two studies presented fluid data, from which intra-operative fluid data was available in 56 studies. However, 4 studies presented these as a rate in ml/kg/h and we were therefore not able to use these for data synthesis. Forty-two studies presented the intra-operative fluid input data separately for colloids and crystalloids. In the GDHT group, there were less crystalloids (−375 ml [95% CI −581 to −170], *I*^2^ = 98%, *P* = 0.0003) and more colloids (+281 ml [95% CI 166 to 396], *I*^2^ = 96%, *P* < 0.00001) administered intra-operatively. There was significant statistical heterogeneity between studies and random-effects models were used for these analyses. Total intra-operative fluid input was presented by 27 studies and the GDHT group overall had less cumulative intra-operative fluid (−212 ml [95% CI −254 to 171], *I*^2^ = 91%, *P* < 0.0001) than controls (Table 4).

**Table 4:** Intraoperative fluid balance. The effect was presented as a mean difference in goal-directed haemodynamic group in comparison with controls. *CI* confidence interval, *IV* inverse variance

Outcome	No. of studies	No. of patients	Analysis	Effect (95% CI)	***P*** value
Intra-operative crystalloids	42	4956	Mean difference (IV, random)	−375 (−581 to −170)	*P* = 0.0003
Intra-operative colloids	42	4984	Mean difference (IV, random)	281 (166 to 396)	*P* < 0.00001
Cumulative intra-operative fluid input	27	2907	Mean difference (IV, random)	−212 (−254 to 171)	*P* < 0.00001

### Subgroup analysis

We carried out a subgroup analysis to assess the impact of GDHT on the incidence of pulmonary complications adjusted for the type of surgery (Table 5). There was a beneficial effect demonstrated for patients undergoing general, mixed or abdominal surgery (OR 0.76 [95% CI 0.58–0.99], *I*^2^ = 54%, *P* = 0.04, 44 studies) and cardiothoracic surgery (OR 0.33 [95% CI 0.17 to 0.63], *I*^*2*^ = 22%, *P* = 0.0009, 7 studies). No reduction in pulmonary complications was observed in patients undergoing orthopaedic (OR 1.15 [95% CI 0.73 to 1.81], *I*^2^ = 0%, *P* = 0.54, 9 studies) or vascular surgery (OR 0.79 [95% CI 0.35 to 1.76], *I*^2^ = 12%, *P* = 0.56, 6 studies).

**Table 5:** Subgroup analysis for the type of surgery, device, the goal-directed haemodynamic therapy intervention (fluids only or fluids and inotropes or vasopressors) and the date when the study was conducted. *CI* confidence interval, *M-H* Mantel-Haenszel, *PAC* pulmonary artery catheter

Subgroups	No. of studies	No. of patients	Analysis	Effect (95% CI)	***P*** value
**Surgery types**					
General/mixed/abdominal	44	7420	M-H odds ratio	0.76 (0.58–0.99)	*P* = 0.04
Orthopaedic	9	900	M-H odds ratio	1.15 (0.73–1.81)	*P* = 0.54
Cardiothoracic	7	797	M-H odds ratio	0.33 (0.17–0.63)	*P* = 0.0009
Vascular	6	431	M-H odds ratio	0.79 (0.35–1.76)	*P* = 0.56
**Device types**					
Minimally invasive	33	3719	M-H odds ratio	0.73 (0.53–1.01)	*P* = 0.06
Oesophageal doppler	17	2003	M-H odds ratio	0.87 (0.43–1.77)	*P* = 0.70
Non-invasive	5	744	M-H odds ratio	0.77 (0.44–1.38)	*P* = 0.38
PAC	8	2688	M-H odds ratio	0.69 (0.44–1.08)	*P* = 0.10
Oxygen indices	3	460	M-H odds ratio	1.13 (0.64–2.00)	*P* = 0.67
**Intervention per protocol**					
Fluids only	22	2033	M-H odds ratio	1.39 (0.89–2.16)	*P* = 0.14
Fluid and inotropes or vasopressors	44	7515	M-H odds ratio	0.62 (0.49–0.80)	*P* = 0.0002
**Date of study publication**					
Conducted up to and year 2000	9	728	M-H odds ratio	0.54 (0.36–0.81)	*P* = 0.003
Conducted between 2001 and 2010	12	3296	M-H odds ratio	0.86 (0.56–1.31)	*P* = 0.48
Conducted between 2011 and to date	45	5524	M-H odds ratio	0.73 (0.54–0.99)	*P* = 0.04

Each study was also categorised according to their method of monitoring. Most studies (50%) used minimally invasive cardiac output monitoring and the results were not significant for any specific category of technique (Table 5).

The GDHT protocol used fluids only in 22 studies. Other studies used a combination of fluid bolus with inotropes and/or vasopressors. Forty-two studies used colloids as their bolus fluid and 9 studies used crystalloids, and a further 9 studies used a combination of both crystalloids and colloids. When studies used fluids in combination with inotropes or vasopressors in the GDHT protocol, there was a significant reduction in pulmonary complications (OR 0.62 [95% CI 0.49 to 0.80], *I*^2^ = 50%, *P* = 0.0002, 44 studies). This was not evident when the protocol used fluid alone (OR 1.39 [95% CI 0.89 to 2.16], *I*^2^ = 17%, P = 0.14, 22 studies) (Table 5). Given the significant advances in perioperative care over the inclusion period of this meta-analysis, we compared the effect of GDHT on pulmonary complications with dates when the studies were conducted. We grouped these trials into 3 cohorts: year 2000 and earlier (OR 0.54 [95% CI 0.36 to 0.81], *I*^2^ = 0%, *P* = 0.003, 9 studies), 2001 to 2010 (OR 0.86 [95% CI 0.56 to 1.31], *I*^2^ = 42%, *P* = 48, 12 studies), and 2011 to the current period (OR 0.73 [95% CI 0.54 to 0.99], *I*^2^ = 53%, *P* = 0.04, 45 studies). Most of the studies were conducted within the current decade (Table 5).

## Discussion

This systematic review and meta-analysis identified 96 studies evaluating the effect of goal-directed fluid therapy, utilising various devices, to assess patients’ outcomes in the perioperative setting. Sixty-six studies with 9548 participants were included in the data synthesis. The overall incidence of pulmonary complication was 13% and, based on moderate-quality evidence, this review shows that peri-operative goal-directed haemodynamic therapy, targeted to augment blood flow by specified measured goals, reduces pulmonary complications. The results are limited by the presence of substantial clinical and moderate statistical heterogeneity. Consequently, we have downgraded the GRADE evidence from high to moderate. This is the first systematic review to assess the impact of goal-directed haemodynamic therapy exclusively on post-operative pulmonary complications in patients undergoing surgery.

The rates of pulmonary infection and pulmonary oedema were significantly lower in the intervention group with a number needed to treat 45 and 48 respectively. Although the data synthesis was limited by publication bias, where only a proportion of published studies reported specific complication rates (pulmonary infections 68%; pulmonary oedema 35%; pulmonary embolism 42%; and ARDS 23%), these findings are consistent with previously published systematic reviews in this area.(Grocott et al., 2012; Chong et al., 2018) To mitigate clinical heterogeneity, we performed subgroup analysis for types of surgery, devices utilised and the GDHT interventions (fluids alone or fluids and inotropes or vasopressors) provided. The beneficial effect was demonstrated in patients undergoing cardiothoracic and major abdominal surgery and was not specific to any device category. The GDHT algorithm with a combination of fluids and vasopressors or inotropes was associated with a significant reduction in post-operative pulmonary complications, whilst no benefit was demonstrated for those using fluids alone.

Although GDHT may impact pulmonary complications through a variety of mechanisms relating to the monitoring device, type of fluid, different combinations of vasopressor and/or inotrope therapy, we found no evidence that this intervention was harmful. The underlying reasons for the demonstrated beneficial effects are not evaluated by this review. However, several possible mechanisms may explain these findings. A GDHT algorithm with a combination of fluids and inotropes or vasopressors seems to have a positive effect on the incidence of pulmonary complications compared with fluid therapy alone. Additionally, the GDHT participants had less crystalloid (380 ml) and more colloid solutions (280 ml) intra-operatively. Whilst these differences are modest, they may in combination have contributed to maintaining intravascular volume, whilst reducing the development of tissue and pulmonary interstitial oedema. The occurrence of a beneficial outcome in patients receiving more colloid during surgery challenges notions that the use of such fluids has no benefit (Chappell et al., 2008). Moreover, goal-directed haemodynamic therapy has been shown to improve gut perfusion(Mythen & Webb, 1995) and mitigate the inflammatory response to surgery(Noblett et al., 2006) as well as improving tissue microvascular flow and oxygenation(Jhanji et al., 2010). Overall, improved tissue perfusion as a result of GDHT may reduce systematic inflammation and thereby minimise secondary pulmonary harm. Future large randomised controlled trials may offer additional information regarding the use of this intervention in emergency and major gastrointestinal surgical patients (ISRCTN14729158 and ISRCTN39653756, Table 1).

Our review has several limitations. We were not able to include all published studies in this area due to a lack of consistent reporting of pulmonary complications. Recent efforts to standardise core outcome sets may improve this situation in the future.(Boney et al., 2016) Furthermore, there was substantial clinical heterogeneity among the studies included in this review. The studies have varied in nature with regard to type and mode of surgery (elective or emergency), devices, protocols and interventions utilised for the goal-directed pathway and were conducted over several decades, during which surgical and anaesthetic practice has evolved substantially (e.g. introduction of laparoscopic surgery and enhanced recovery). Some studies were conducted in high-risk groups and others embedded enhanced recovery programmes (ERAS). We did not differentiate between these as they were not specifically reported by all studies. We have used author-defined definitions for pulmonary complications such as atelectasis, pulmonary infections or pneumonia, ALI and ARDS. Reporting of such definitions was variable between studies and may have introduced bias. We have included all patients with pulmonary oedema and whilst this was inclusive of patients with respiratory causes of pulmonary oedema it may have introduced additional bias from the inclusion of patients with cardiogenic pulmonary oedema. However, we did exclude studies that specifically mentioned pulmonary oedema of cardiogenic origin. Despite all these limitations, we have included as many of the studies as possible that reported pulmonary complications. We were also unable to include other robust clinical outcomes such as intensive care utilisation and the requirement for ventilatory support resulting from post-operative pulmonary complications due to a lack of consistent reporting. We have included both studies that augment haemodynamic variables to normal levels, as well as those that aimed to achieve supra-normal values of oxygen delivery. Recently, several studies have aimed to restrict fluid volume as part of their GDHT targets; however, we did not separate these in our data synthesis.

The paucity of clinical data and lack of consistent reporting of fluid administration among studies may have introduced publication bias, where not all the studies reporting the intra-operative fluid data in a format that we could analyse. As a result, we have performed data transformation to generate standard deviation and mean values from the median data presented (interquartile range, range, 95% confidence intervals, and 25th and 75th percentiles), guided by the Cochrane Collaboration. Furthermore, most studies reported intra-operative fluid volumes separately for crystalloids and colloids. Some studies reported these as ml/kg/hour and others as a cumulative balance, or total volumes used. Despite these limitations, it appears that in the GDHT group, more colloid and less crystalloid fluids were used during the intraoperative period. Most of the studies (63%) utilised a colloid solution as their fluid of choice in their protocol for fluid bolus regimens and this is the likely explanation for the increased usage of colloids. Others used a combination of crystalloids and colloids (14%), or crystalloids alone (14%) and in six studies the type of fluid was not specified.

The risk of bias was an issue among the included studies. Many of the studies (62%) included in this review did not blind the participants or personnel delivering the intervention peri-operatively and as a result, may have increased the risk of performance bias. Although some studies have attempted to blind but ultimately have found it difficult to fully blind the clinicians involved in delivering the therapy. Taking a pragmatic view, it is difficult to blind clinicians performing a continuous intervention that requires feedback and action in real-time and this has been taken into consideration when performing our risk of bias assessment and the interpretation of the results. Overall, the GRADE quality of evidence was graded as moderate due to substantial clinical and moderate statistical heterogeneity with a high risk of bias in several included studies.

Despite these limitations, the results of this systematic review and meta-analysis add to a growing body of literature suggesting that GDHT is safe and is effective in reducing the burden of a wide range of perioperative outcomes, in this case, respiratory complications. Whilst clinical adoption of GDHT is recognised to be variable, as the evidence base continues to grow the signal of benefit remains consistent. Understandable concerns about the evidence base are primarily focussed on the clinical heterogeneity, particularly the long duration of time over which these studies have taken place and the associated changes in clinical practice that have occurred, potentially limiting the validity of the findings for current practice. Results from two large (>2000 patient) randomised controlled trials currently ongoing may clarify this picture soon.(Edwards et al., 2019; http://www.isrctn.com/ISRCTN14729158, n.d.)

## Conclusions

This study has shown a reduction in post-operative pneumonia and pulmonary oedema following the use of GDHT. These beneficial effects were seen in studies using fluids in combination with inotropes or vasopressors in the GDHT protocol, but not when the protocol used fluid alone. Sub-group analyses demonstrated (i) benefit from GDHT in general/abdominal/mixed and cardiothoracic surgery but not in orthopaedic and vascular surgery; and (ii) no difference between devices used in the GDHT protocols. There was no demonstrable difference in the total volumes of fluid administered but there was greater use of colloid and less of crystalloid solutions in the GDHT group.

## Supplementary information

**Additional file 1: Supplementary material Table.** A Study characteristics in detail. Supplementary material Figure A: review authors’ judgements about each risk of bias item presented as percentages across all included studies. Green indicates no risk of bias, yellow and red represents unclear risk and high risk respectively

## Data Availability

The datasets during and/or analysed during the current study available from the corresponding author on reasonable request.
