# Supplementary material for: Goal-directed haemodynamic therapy (GDHT) in surgical patients: systematic review and meta-analysis of the impact of GDHT on post-operative pulmonary complications
Source: Perioper Med (Lond). 2020 Oct 15;9:30. doi: 10.1186/s13741-020-00161-5 (PMC7560066; doi:10.1186/s13741-020-00161-5)
Supplement: Supplementary file 1 — Additional file 1: Supplementary material Table. A Study characteristics in detail. Supplementary material Figure A: review authors’ judgements about each risk of bias item presented as percentages across all included studies. Green indicates no risk of bias, yellow and red represents unclear risk and high risk respectively [file 13741_2020_161_MOESM1_ESM.docx]

**Table A**: Study characteristics in detail

| Study | Methods | Patient Risk | Participants | Interventions | Outcomes | Notes |
| --- | --- | --- | --- | --- | --- | --- |
| Ackland 2015  UK | RCT  Elective major surgery  Multi-centre | High | **204 Randomised**  102 GDT  102 Control  **Analysed**  95 GDT (no intervention =6, GDT discontinued =1)  92 Control (no intervention =10) | **Monitoring**:  Minimally invasive cardiac output monitor  **Target**:  SV, CO  **Therapy**:  Fluids + Inotropes/vasopressors  **Timing:**  Post-operative | **Primary**:  Absolute RR of acquiring post-operative morbidity  **Secondary**:  Time to become morbidity free; hospital LoS | Several authors have industry affiliations |
| Bahlmann 2019  Sweden | RCT  Elective transthoracic oesophageal resection  Multi-centre | Low  (ASA 1-3, with <1/3 of pts ASA 3) | **64 Randomised**  32 GDT  32 Control  **Analysed**  30 GDT (surgery cancelled/aborted =2)  29 Control (surgery aborted =3) | **Monitoring**:  Minimally invasive cardiac output monitor  **Target**:  SV maximisation, CI ≥2.5L/min, MAP ≥65mmHg  **Therapy**:  Fluids + inotropes/vasopressors  **Timing:**  Intra-operative  Post-operative | **Primary**:  Complications at 5 and 30 days  **Secondary**:  Length ICU stay; length hospital stay; time to pass flatus; long term survival (added later); fluid balance; body weight changes; intra-operative catecholamine use | Slow recruitment therefore long term survival added as secondary outcome  Sponsored by Linkoping Medical Society |
| Bartha 2013  Sweden | RCT  Elective proximal femoral fracture repair during regular operating hours  Single centre | High  (>50% ASA 3 or 4) | **150 randomised**  74 GDT (1 patient enrolled twice)  75 Control  **Analysed**  70 GDT (did not receive treatment = 4)  72 control (failed art line =2, tech error =1) | **Monitoring**:  Minimally invasive cardiac output monitor  **Target**:  SV maximisation, DO_2_I >600  **Therapy**:  Fluids + Inotropes/vasopressors  **Timing**:  Pre-operative  Intra-operative | **Primary**:  Absolute RR of post-operative complications.  **Secondary**:  Volume of fluids administered; intra-operative hypotension; intra-operative haemodynamic response; 30 day mortality;  aggregated healthcare costs; use of social services; post-operative QoL at 12 months | Funding: Stockholm county grant. |
| Bender 1997  USA | RCT  Elective infrarenal aortic reconstruction or lower limb revascularisation  Single centre | High  (most patients ASA≥3) | **104 randomised**  51 GDT  53 Control  **All analysed** | **Monitoring:**  PAC  **Target**:  CI≥2.8, PAOP 8-14, SVR ≤1100  **Therapy**:  Fluids + Inotropes/vasopressors  **Timing**:  Pre-operative  Intra-operative  Post-operative | **Primary**:  Mortality  **Secondary**: Post-operative complications; ICU LoS; hospital LoS | No conflict of interest declared. |
| Benes 2010  Czech Republic | RCT  Elective intra-abdominal surgery  Single centre | High  (One or more of: surgery ≥120min with opened peritoneal cavity; EBL >1L, AND one or more of: IHD or severe heart dysfunction, COPD, Age≥70, ASA≥3) | **120 randomised**  60 GDT  60 Control  **Analysed**  60 GDT (post-operative outcome)  51 GDT (intra-operative + biochemistry)  60 Control (post-operative outcome)  54 Control (intra-operative + biochemistry) | **Monitoring**:  Minimally invasive cardiac output monitor  **Target**:  SVV <10%, CI 2.5-4  **Therapy**:  Fluids + Inotropes/vasopressors  **Timing**:  Intra-operative | **Primary**:  Post-operative morbidity at 30 days  **Secondary**:  Hospital LoS; ICU LoS; all-cause mortality | Research grant from the Czech Ministry of Education |
| Bisgaard 2013 (1)  Denmark | RCT  Elective open abdominal aortic surgery  Single centre | High  (>50% ASA≥3) | **70 randomised**  35 GDT  35 Control  **Analysed**  32 GDT (technical/re-operation/surg abandoned = 3)  32 Control (technical/surg abandoned = 3) | **Monitoring**:  Minimally invasive cardiac output monitor  **Target**:  SVI >10% with fluid boluses, DO_2_I >600  **Therapy**:  Fluids + Inotropes/vasopressors  **Timing:**  Intra-operative  Post-operative | **Primary**:  Number of post-operative complications  **Secondary**:  ICU LoS; hospital LoS | (1) Lillebaelt Hospital Kolding (Denmark) – Local research fund.  (2) The Toyota Fund (Denmark).  (3) Research Initiative of the Danish Society of Anaesthesiology and Intensive Care Medicine (Denmark). |
| Bisgaard 2013 (2)  Denmark | RCT  Elective lower limb arterial surgery  Single centre | High  (75% ASA≥3) | **40 randomised**  20 GDT  20 Control  **All analysed** | **Monitoring**:  Minimally invasive cardiac output monitor  **Target**:  SVI >10% with fluid boluses, DO_2_I >600  **Therapy**:  Fluids + Inotropes/vasopressors  **Timing**:  Intra-operative  Post-operative | **Primary**:  Incidence of post-operative complications  **Secondary**:  Haemodynamic values; hospital LoS | (1) Lillebaelt Hospital Kolding (Denmark) – Local research fund.  (2) The Toyota Fund (Denmark).  (3) Research Initiative of the Danish Society of Anaesthesiology and Intensive Care Medicine (Denmark). |
| Boyd 1993  UK | RCT  Mixed surgical population. Mixed elective and emergency.  Single centre | High | **107 randomised**  53 GDT  54 Control  **All analysed** | **Monitoring**:  PAC  **Target**:  GDT group: DO_2_I >600  **Therapy**:  Fluids + Inotropes/vasopressors  **Timing**:  Pre-operative  Intra-operative  Post-operative | **Primary**:  Mortality at 28 days;  morbidity  **Secondary**:  Post-operative complications; ICU LoS; hospital LoS | Grant from Fisons PLC, UK. |
| Brandstrup 2012  Denmark | RCT  Elective colorectal patients  Multi-centre | Low  (majority ASA 1-2) | **151 randomised**  72 GDT  79 Control  **Analysed**  71 GDT (cancelled surgery =1)  79 Control | **Monitoring**:  OD  **Target**:  SV maximisation (SV increase <10%)  **Therapy**:  Fluids + inotropes/vasopressors  **Timing**:  Intra-operative | **Primary**:  Post-operative complications and mortality combined  **Secondary**:  Hospital LoS; antiemetic use; diuretic use; physiological change; vasopressor requirements | This trial was funded by Aase and Einar Danielsen’s Fund. |
| Calvo-Vecino 2018  Spain | RCT  Mixed elective major surgery.  Multi-centre | Low  (majority ASA 1-2) | **450 randomised**  224 GDT  226 Control  **420 analysed**  209 GDT (did not receive allocated surgery = 10, orthopaedic therefore excluded = 5)  211 Control (did not receive allocated surgery = 12, orthopaedic therefore excluded = 3) | **Monitoring**:  OD  **Target**:  SV maximisation with fluid boluses, CI >2.5  **Therapy**:  Fluids + inotropes/vasopressors  **Timing**:  Intra-operative | **Primary**:  Percentage of patients who developed complications within 180 days.  **Secondary**:  Hospital LoS; ICU LoS; re-intervention rate; time to oral tolerance and ambulatory; all-cause mortality at 180 days | Several authors have industry affiliations.  The trial was supported by Unidad de Evaluación de Tecnologías Sanitarias, Madrid, Spain. |
| Cecconi 2011  Italy | RCT  Elective orthopaedic  Single centre | Low  (majority ASA 2) | **40 randomised**  20 GDT  20 Control  **All analysed** | **Monitoring:**  Minimally invasive cardiac output monitor  **Target**:  SV maximisation with fluid boluses, DO_2_I >600  **Therapy**:  Fluids + inotropes/vasopressors  **Timing**:  Intra-operative  Post-operative | **Primary**:  Mortality; Post-operative complications  **Secondary**:  Post-operative nausea and vomiting; hospital LoS | Two authors received lecturing fees from Edwards Lifesciences, LiDCO and Cheetah Medical, as well as research support from LiDCO. |
| Colantonio 2015  Italy | RCT  Elective peritoneal carcinomatosis candidates for peritonectomy and HIPEC  Single centre | Low  (majority ASA 2) | **86 randomised**  42 GDT  44 Control  **Analysed**  38 GDT (hipec cancelled =3, anaesthetic complication =1)  42 Control (hipec cancelled =1, anaesthetic complication =1) | **Monitoring**:  Minimally invasive cardiac output monitor  **Target**:  CI >2.5, SVI>35ml/m^2^,  **Therapy**:  Fluids + inotropes/vasopressors  **Timing**:  Intra-operative | **Primary**:  Major abdominal complications at 30 days  **Secondary**:  Systemic complications at 30 days; hospital LoS; 30 day mortality; ICU readmission rate | Departmental funding only |
| Correa-Gallego 2015  USA | RCT  Elective open liver resections  Single centre | Low  (majority ASA 1-2) | **135 randomised**  69 GDT  66 Control  **All analysed** | **Monitoring**:  Minimally invasive cardiac output monitor  **Target**: SVV to within 2 st.deviations of baseline  **Therapy**:  Fluids only  **Timing**:  Intra-operative | **Primary**:  30 day mortality  **Secondary**:  30 day grade 3 or greater morbidity; total fluid volume administered; vasopressor use; blood transfusion rate;  dnd-organ perfusion; time to reach post-operative recovery milestones | No conflict of interest declared. |
| Davies 2019  UK | RCT  Emergency repair of proximal femoral fracture  Multi-centre | High  (majority ASA 3 or 4) | **241 randomised**  121 GDT  120 Control  **Analysed**  120 GDT (protocol violation = 1)  120 control | **Monitoring**:  Non-invasive cardiac output monitor  **Target**:  SV maximisation  **Therapy**:  Fluids + inotropes/vasopressors  **Timing**:  Intra-operative | **Primary**:  Number of patients who developed one or more in-hospital post-operative complication  **Secondary**:  Hosp LOS; POMS morbidity day 3, day 5, day 10; intra-operative haemodynamic variables; volume of intra-operative fluid, intra-operative hypotension, intra-operative vasopressors. | Sponsored by Ewards Lifesciences |
| Donati 2007  Italy | RCT  Elective abdominal surgery  Multi-centre | High | **135 randomised**  68 GDT  67 Control  **All analysed** | **Monitoring**:  Venous oxygen saturations  **Target**:  O_2_ER ≤27%  **Therapy**:  Fluids + inotropes/vasopressors  **Timing**:  Intra-operative  Post operative | **Primary**:  Number of patients with one new organ dysfunction post-operatively  **Secondary**:  Number of organ failures during ICU stay; hospital LoS; mortality | No conflict of interest declared. |
| El-Sharkawy 2013  Egypt | RCT  Elective liver resection  Single centre | Low | **59 randomised**  29 GDT  30 Control  **Numbers analysed not specified but only 39 pts appear in demographics table** | **Monitoring:**  OD  **Target**:  SV maximisation with fluid boluses, FTc >0.35s  **Therapy**:  Fluids only  **Timing**:  Intra-operative  Post-operative | **Primary**:  Perioperative morbidity  **Secondary**:  Hospital LoS | No conflict of interest declared. |
| Funk 2015  Canada | RCT  Elective AAA repair  Single centre | High  (mean P-Possum mortality ≥8%) | **40 randomised**  20 GDT  20 Control  **All analysed** | **Monitoring**:  Minimally invasive cardiac output monitor  **Target**:  SVV <13% with fluid boluses, CI >2.2 with inotropes  **Therapy**:  Fluids + inotropes/vasopressors  **Timing**:  Intra-operative | **Primary**:  Post-operative inflammatory marker levels  **Secondary**:  Hospital LoS; post-operative complications | Funded by Winnipeg Health Sciences Centre Research Foundation |
| Gan 2002  USA | RCT  Elective mixed major abdominal surgery  Single centre | Low  (majority ASA 1-2) | **100 randomised**  50 GDT  50 Control  **Analysed**  49 GDT (no operation = 1)  49 Control (no operation = 1) | **Monitoring**:  OD  **Target**:  SV maximisation, FTc >0.35s  **Therapy**:  Fluids only  **Timing**:  Intra-operative | **Primary**:  Hospital LoS  **Secondary**:  Post-operative complications; haemodynamic variables; intra-operative fluid and blood product administration | No conflict of interest declared. |
| Gerent 2018  Brazil | RCT  Elective high risk abdominal surgery  Single centre | High  (all pts required ICU admission) | **128 randomised**  64 GDT  64 Control  **All analysed** | **Monitoring**:  Minimally invasive cardiac output monitor  **Target**:  SVI >35, CI ≥2.5  **Therapy**:  Fluids + inotropes/vasopressors  **Timing:**  Post-operative | **Primary**:  30 day mortality; major clinical complications whilst in hospital  **Secondary**:  Incidence of septic shock; development of AKI requiring RRT; ICU readmission rate; ICU LoS; hospital LoS; Day 7 SOFA score; 90 day mortality | The two funding sources were the University of Sao Paulo (Brazil) and Edwards LTDA (Irvine, CA, USA), which had no other role in the study. |
| Goepfert 2013  Germany | RCT  Elective cardiac surgery  Single centre | High  (all ASA 3-4) | **100 randomised**  50 GDT  50 Control  **All analysed** | **Monitoring**:  Minimally invasive cardiac output monitor  **Target**:  SVV, CI, GEDI  **Therapy**:  Fluids + inotropes/vasopressors  **Timing**:  Intra-operative  Post-operative | **Primary**:  Duration of ICU therapy  **Secondary**:  Need for vasoactive drugs; Incidence of post-operative complications | Unrestricted grant from Pulsion Medical Systems AG, Munich, Germany. |
| Gómez-Isquierdo 2017  Canada | RCT  Elective laparoscopic resection  Single centre | Low  (majority ASA 1-2, average P-POSSUM mortality <2%) | **135 randomised**  68 GDT  67 Control  **Analysed (ITT)**  64 GDT (withdrew consent = 1, drop-out, carcinomatosis = 2, discontinued intervention – cannula fault = 1)  64 Control (change of surgical pre-operatively –lap to open =1, dropout, carcinomatosis = 2)  **Analysed (per protocol)**  56 GDT (excluded – open = 8)  59 Control (excluded – open = 5) | **Monitoring**:  OD  **Target**:  SV maximisation with fluid boluses  **Therapy:**  Fluids only  **Timing**:  Intra-operative | **Primary**:  Incidence of post-operative ileus  **Secondary**:  Quality of recovery score; 30 day complications; readiness to be discharged; hospital LoS; readmission rates | Supported by the Gerard R. Douville Award, granted to Dr. Baldini, McGill University Health Center Research Institute, Montreal, Quebec, Canada. |
| Han 2016  China | RCT  Elective THR  Single centre | Low  (ASA 1-2) | **40 randomised**  20 GDT  20 Control  **All analysed** | **Monitoring**:  Minimally invasive cardiac output monitor  **Target**:  SVV <10%  **Therapy**:  Fluids only  **Timing**:  Intra-operative | **Primary**:  Haemodynamic changes (HR, MAP,  lactate level, urine output, phenylephrine use)  **Secondary**  Post-operative complications | No conflict of interest declared. |
| Jammer 2010  Norway | RCT  Elective open bowel surgery  Multi-centre | Low  (majority ASA 1-2) | **241 randomised**  121 GDT  120 Control  **All analysed** | **Monitoring**:  Central venous oxygen saturations  **Target**:  ScVO_2_  **Therapy**:  Fluids only  **Timing**:  Intra-operative  Post operative | **Primary**:  Post-operative complications at 30 days  **Secondary**:  Post-operative serum creatinine; SpO_2_; weight | No conflict of interest declared. |
| Jammer 2015  Norway & Finland | RCT  Open major abdominal surgery  Multi-centre | High  (ASA 3-4) | **30 randomised**  14 GDT  16 Control  **All analysed** | **Monitoring**:  Minimally invasive cardiac output monitor  **Target**:  SVV  **Therapy**:  Fluids + inotropes/vasopressors  **Timing**:  Intra-operative | **Primary**:  Post-operative complications at 5 days  **Secondary**:  Renal function (RIFLE); lactate, BE, PaO_2_; urine output; hospital LoS; complications until discharge; readmission rate at 30 days; utilisation of vasoactive drugs; 30 day and 3 month mortality | This study was supported by an unrestricted grant by The Eckbo Foundations, Norway and departmental funding. |
| Joosten 2019  Belgium | RCT  Elective mixed surgery  Single centre | Low  (ASA 1-3) | **40 randomised**  **Analysed**  20 GDT  19 Control  (excluded due to anaphylaxis = 1) | **Monitoring**:  Non-invasive cardiac output monitor  **Target**:  SV optimisation using closed loop software (CI ≥2.5, SVV <13%)  **Therapy**:  Fluids only  **Timing**:  Intra-operative | **Primary**:  Percentage of intra-operative time spent within haemodynamic targets  **Secondary**:  Time-in-target for separate CI and SVV components of primary outcome; total fluid volume; fluid balance; post-operative complications within 30 days; PACU LoS; hospital LoS | Several authors have industry affiliations; two authors own patents on closed loop fluid management systems. |
| Kaufmann 2017  Germany | RCT  Elective thoracic surgery  Single centre | High  (Majority ASA 3) | **100 randomised**  50 GDT  50 Control  **Analysed**  48 GDT (blood loss >1.5L = 1, no parenchymal resection = 1)  48 Control (blood loss 1.5L = 2) | **Monitoring**:  OD  **Target**:  SV maximisation with fluid boluses, CI >2.5, MAP >70  **Therapy:**  Fluids + inotropes/vasopressors  **Timing**:  Intra-operative | **Primary**:  In-hospital post-operative pulmonary complications  **Secondary**:  AKI; cardiac morbidity; clinically relevant hypotensive episodes; neurological morbidity; pleural effusion; pain intensity; hospital LoS | This work was supported by departmental funding. |
| Kaufmann 2018  Germany | RCT  Mixed elective and emergency orthopaedic surgery  Single centre | High  (Majority ASA 3) | **90 randomised**  45 GDT  45 Control  **All analysed** | **Monitoring**:  OD  **Target**:  SV maximisation with fluid boluses, CI >2.5, MAP >70  **Therapy:**  Fluids + inotropes/vasopressors  **Timing:**  Intra-operative | **Primary**:  Incidence of bone cement implantation syndrome  **Secondary**:  Cardiac function; hospital LoS; post-operative complications | This work was supported by departmental funding. |
| Kim 2018  South Korea | RCT  Elective head and neck surgery  Single centre | Low  (Majority ASA 1-2) | **62 Randomised**  31 GDT  31 Control  **All analysed** | **Monitoring:**  Minimally invasive cardiac output monitor  **Target**:  SVV <12%, CI ≥2.5, MAP ≥65  **Therapy:**  Fluid + inotropes/vasopressors  **Timing**:  Intra-operative | **Primary**:  Hospital LoS  **Secondary**:  ICU LoS; flap condition; reoperation rate; post-operative complications; cytokine levels | No conflict of interest declared. |
| Kumar 2016  India | RCT  Elective major abdominal surgery  Single centre | Low  (ASA 1-2) | **60 randomised**  30 GDT  30 Control  **All analysed** | **Monitoring**:  Minimally invasive cardiac output monitor  **Target**:  SVV <10%, SVR, MAP >65  **Therapy**:  Fluids + inotropes/vasopressors  **Timing**:  Intra-operative | **Primary**:  ICU LoS; hospital LoS  **Secondary**:  Intra-operative lactate; IV fluid use; requirement for vasoactive medications; post-operative ventilation; time to return of bowel function | No conflict of interest declared. |
| Lobo 2000  Brazil | RCT  Elective major abdominal surgery  Single centre | High  (ICU patients) | **37 randomised**  19 GDT  18 Control  **All analysed** | **Monitoring**:  PAC  **Target**:  DO_2_I (control 520-600, GDT >600) **Therapy**:  Fluids + inotropes/vasopressors  **Timing**:  Intra-operative  Post-operative | **Primary**:  60 day mortality  **Secondary**:  Post-operative complications; organ dysfunction | No conflict of interest declared. |
| Luo 2017  China | RCT  Elective neurosurgery  Single centre | High  (ASA 3-4) | **150 randomised**  75 GDT  75 Control  **Analysed**  73 GDT  72 Control  (withdrew consent = 5) | **Monitoring**:  Minimally invasive cardiac output monitor  **Targets**:  SVV, CI  **Therapy**:  Fluids + inotropes/vasopressors  **Timing:**  Intra-operative | **Primary**:  ICU LoS  **Secondary**:  Lactate at the end of surgery; post-operative complications at 30 days; post-operative morbidity at 30 days; 30 day mortality; hospital LoS; cost of admission | Supported by the Science and Technology Dept of Sichaun Province support program. |
| Mayer 2010  Germany | RCT  Elecive high risk abdominal surgery  Single-centre | High  (ASA 3 + ≥2 high risk features) | **60 randomised**  30 GDT  30 Control  **All analysed** | **Monitoring**:  Minimally invasive cardiac output monitor  **Targets**:  CI≥2.5, SVI >35  **Therapy**:  Fluids + inotropes/vasopressors  **Timing**:  Intra-operative | **Primary**:  Hospital LoS  **Secondary**:  Incidence of post-operative outcomes; ICU LoS; fluid use; inotrope/vasopressor use | Unrestricted grant from Edwards Lifesciences, USA |
| McKendry 2004  UK | RCT  Mixed elective and emergency cardiothoracic surgery  Single centre | High  (Cardiac surgery) | **179 randomised**  89 GDT  90 Control  **Analysed**  89 GDT  85 control (did not receive intervention as too unstable = 5) | **Monitoring**:  OD  **Targets**:  SVI >35  **Therapy**:  Fluids + inotropes/vasopressors  **Timing**:  Post-operative | **Primary**:  Hospital LoS; ICU LoS  **Secondary**:  Incidence of post-operative complications | Deltex provided an unrestricted educational grant for this study, and funds to the department and research nurses. |
| McKenny 2013  Ireland | RCT  Elective major open gynaecological surgery  Single centre | Low  (Majority ASA 2) | **102 randomised**  51 GDT  51 Control  **Analysed**  51 GDT  50 Control (did not receive intervention = 1) | **Monitoring**:  OD  **Targets**:  SV  **Therapy**:  Fluids only  **Timing**:  Intra-operative | **Primary**:  Time until medically fit for discharge  **Secondary**:  Time to tolerate oral intake; time to bowels open; POMS Score; incidence of wound infections, renal dysfunction, pneumonia and unplanned ICU admission | No conflict of interest declared. |
| Mikor 2015  Hungary | RCT  Elective major abdominal surgery  Single centre | High  (All patients admitted to ICU post-operatively) | **84 randomised**  42 GDT  42 Control  **Analysed**  38 GDT (did not receive intervention = 4)  41 Control (did not receive intervention = 1) | **Monitoring**:  Central venous oxygen saturations  **Target**:  ScVO_2_ 75%, MAP >60  **Therapy**:  Fluids + inotropes/vasopressors  **Timing**:  Intra-operative | **Primary**:  Incidence of post-operative complications by day 2  **Secondary**:  Intra-operative interventions | One author is a member of PULSION medical advisory board and receives honoraria for lectures. |
| Moppett 2015  UK | RCT  Hip fracture repair  Single centre | High | **130 randomised**  62 GDT  68 Control  **Analysed**  51 GDT (did not receive intervention = 11)  63 Control (did not receive allocated intervention = 5) | **Monitoring**:  Minimally invasive cardiac output monitor  **Target**:  SV optimisation with fluid boluses  **Therapy**:  Fluids only  **Timing**:  Intra-operative | **Primary**:  Time until medically fit for discharge  **Secondary**:  Acute ward LoS; hospital LoS; incidence of post-operative complications; post-operative mortality | This trial was funded by the National Institute for Health Research (NIHR). |
| Mythen 1995  UK | RCT  Elective cardiac surgery  Single centre | High  (ASA 3 with LVEF >50%) | **60 randomised**  30 GDT  30 Control  **All analysed** | **Monitoring:**  OD  **Target**:  SV maximisation with fluid boluses  **Therapy**:  Fluids only  **Timing:**  Intra-operative | **Primary**:  Gut mucosal pH  **Secondary**:  Incidence of major complications; hospital LoS; ICU LoS; inpatient mortality | Funding from Sir Jules Thorn Charitable Trusts, Leopold Pharma, and Oxford Nutrition |
| Osawa 2016  Brazil | RCT  Elective cardiac surgery  Single centre | High risk | **126 randomised**  62 GDT  64 Control  **All analysed** | **Monitoring**:  Minimally invasive cardiac output monitor  **Targets**:  CI >3, SVI >35  **Therapy**:  Fluids + inotropes/vasopressors  **Timing**:  Intra-operative  Post-operative | **Primary**:  Mortality at 30 days;  major post-operative complications  **Secondary**:  Incidence of delirium, seizure, AKI, VTE, arrhythmia at 30 days; ICU LoS; hospital LoS; daily SOFA Score | Several authors declared industry funding. |
| Pearse 2005  UK | RCT  Mixed elective and emergency major general surgery  Singe centre | High risk | **122 randomised**  62 GDT  60 Control  **All analysed** | **Monitoring**: Minimally invasive cardiac output monitor  **Targets**:  SV optimisation with fluid boluses, DO_2_I >600  **Therapy**:  Fluids + inotropes/vasopressors  **Timing**:  Post-operative | **Primary**:  Incidence of post-operative complications  **Secondary**:  Hospital LoS; mortality | Several authors declared industry funding. |
| Pearse 2014  UK | RCT  Mixed elective and emergency major gastrointestinal surgery  Multi-centre | High risk | **734 randomised**  368 GDT  366 Control  **Analysed**  366 GDT (withdrew consent = 2)  364 control (randomised in error = 1, withdrew consent = 1) | **Monitoring**:  Minimally invasive cardiac output monitor  **Targets**:  SV optimisation with fluid boluses and fixed rate dopexamine  **Therapy**:  Fluids + inotropes/vasopressors  **Timing**:  Intra-operative  Post-operative | **Primary**:  Relative risk of mortality and complications at 30 days  **Secondary**:  Morbidity at day 7; infectious complications; number of critical care-free days; mortality at 30 days; mortality at 180 days; hospital LoS | Several authors declared industry funding. |
| Peng 2014  China | RCT  Elective major orthopaedic surgery  Single centre | Low  (Majority ASA 1 or 2) | **80 randomised**  40 GDT  40 Control  **All analysed** | **Monitoring**:  Minimally invasive cardiac output monitor  **Target**:  SVV ≤10% (supine) or ≤14% (prone)  **Therapy**:  Fluids only  **Timing**:  Intra-operative. | **Primary**:  Time to passage of first flatus  **Secondary**:  Post-operative complications; fluid management; hospital LoS; mortality | No conflict of interest declared. |
| Pestaña 2014  Spain | RCT  Elective open colorectal, gastrectomy or small bowel resection  Multi-centre | High | **170 randomised**  85 GDT  85 Control  **Analysed**  72 GDT ITT (change in surgical procedure = 10, not admitted to ICU = 3)  70 Control ITT (change in surgical procedure = 9, not admitted to ICU = 6) | **Monitoring**:  Non-invasive cardiac output monitor  **Targets**:  MAP ≥65, CI ≥2.5, SV optimisation with FB  **Therapy**:  Fluids + inotropes/vasopressors  **Timing**:  Intra-operative | **Primary**:  Hospital LoS; morbidity  **Secondary**:  Time to first flatus; incidence of wound infection; incidence of anastomotic leak; mortality | The monitors and electrode stickers required for the study were provided by Cheetah Medical. None of the investigators received any economic wages or compensation. |
| Phan 2014  Australia | RCT  Elective major colorectal surgery  Single centre | Low  (Median ASA 2) | **100 randomised**  50 GDT  50 Control  **All analysed** | **Monitoring**:  OD  **Targets**:  SVI >35, FTc >360ms  **Therapy:**  Fluids only  **Timing**  Intra-operative | **Primary**:  Hospital LoS  **Secondary**:  Incidence of post-operative complications; volume of IV fluids administered; changes in haemodynamic parameters | This study was supported by a St Vincent’s Hospital Research Endowment Fund 2012, AUD $20,000. |
| Reisinger 2017  Holland | RCT  Elective colorectal resection  Single centre | Low  (Majority ASA 2) | **58 randomised**  27 GDT  31 Control  **All analysed** | **Monitoring**:  OD  **Targets:**  SVI  **Therapy**:  Fluids + inotropes/vasopressors  **Timing:**  Intra-operative  Post-operative | **Primary**:  Intestinal epithelial damage  **Secondary**:  Pr-aCO_2_ gap; haemodynamic parameters | The study was funded by a Stichting Sint Annadal scholarship, Maastricht University Medical Centre, 2009. |
| Salzwedel 2013  Germany, Russia, Hungary, Spain | RCT  Elective abdominal surgery (including general, gynaecological, urological)  Multi-centre | Low  (majority ASA 1 or 2) | **180 randomised**  (20 excluded)  **Analysed**  79 GDT  81 Control | **Monitoring**:  Minimally invasive CO monitor  **Targets**:  PPV <10%, CI ≥2.5, MAP >65  **Therapy**:  Fluids + inotropes/vasopressors  **Timing**:  Intra-operative | **Primary**:  Post-operative complications up to day 28  **Secondary**:  Hospital LoS | Study funded by a PULSION research grant; several authors are board members of PULSION medical |
| Sandham 2003  Canada | RCT  Mixed urgent and elective major abdominal, thoracic, vascular, hip fracture surgery  Multi-centre | High  (ASA 3 or 4) | **1,994 randomised**  997 GDT  997 Control  **Analysed (for post op complications)**  941 GDT  965 Control | **Monitoring**:  PAC  **Targets**:  MAP ≥70, DO_2_I 550-600, CI 3.5-4.5, PAOP <18, HR <120, Hct >27%  **Therapy**:  Fluids + inotropes/vasopressors  **Timing:**  Pre-operative | **Primary**:  In-hospital mortality  **Secondary**:  6-month mortality; 12-month mortality; in-hospital morbidity | Supported by grants from the Canadian Institute for Health Research and Abbott Laboratories of Canada. |
| Scheeren 2013  Germany | RCT  Elective high risk surgical patients, general and urological.  Multi-centre | High risk  (ASA 3 or 4) | **64 randomised**  32 GDT  32 Control  **Analysed**  26 GDT (protocol violation = 4, arrhythmia = 1, BMI >40 = 1)  26 Control (protocol violation = 4, arrhythmia = 1, BMI >40 = 1) | **Monitoring**:  Minimally invasive cardiac output monitor  **Targets**:  SVV <10%  **Therapy**:  Fluids only  **Timing**:  Intra-operative | **Primary**:  Incidence of post-operative complications  **Secondary**:  SOFA Score; TISS-28 Score; duration of mechanical ventilation; ICU LoS  ICU Discharge criteria  28 day mortality | The work was supported by Edwards Lifesciences Corporation. All authors received honoraria for lectures from Edwards Lifesciences. |
| Schmid 2016  Germany | RCT  Major abdominal surgery  Single centre | High  (expected ICU stay >3 days) | **193 Randomised**  95 GDT  98 Control  **Analysed**  GDT 92 (did not receive allocated intervention = 3)  Control 88 (did not receive allocated intervention = 10) | **Monitoring**:  PICCO  **Targets**:  CI >2.5, MAP >70, GEDI >640, ELWI <10  **Therapy:**  Fluids + inotropes/vasopressors  **Timing**:  Intra-operative  Post-operative | **Primary**:  Maximum change in serum creatinine and creatinine clearance within 7 days after operation  **Secondary**:  RIFLE score in ICU; incidence of AKI; need for dialysis; surgical re-intervention; respiratory and cardiocirculatory complications; sepsis; post-operative mortality. | Funded by institutional support. |
| Senagore 2009  USA | RCT  Elective laparoscopic segmental colectomy  Single centre | Low  (ASA 1-3) | **64 randomised**  42 GDT  22 Control  **All analysed** | **Monitoring**:  OD  **Targets**:  SV optimisation with fluid boluses  **Therapy**:  Fluids only  **Timing**:  Intra-operative | **Primary**:  Hospital LoS  **Secondary**:  Intraoperative fluid requirement; number of days to liquid diet, solid diet, opening bowels; incidence of complications; type of fluid received | Supported by an unrestricted educational grant from Deltex Medical, Inc., Irving, Texas. |
| Shoemaker 1988  USA | Series 2 is an RCT  Mixed elective and emergency high risk general surgical patients  Single centre | High risk | **88 randomised**  28 GDT (supranormal)  30 GDT (normal)  30 Control (CVP)  **All analysed** | **Monitoring**:  PAC  **Targets**:  GDT supranormal: CI >4.5, DO_2_>600, VO_2_>170  GDT normal: CI 2.8-3.5, DO_2_ 400-550, VO_2_ 120-140  **Therapy**:  Fluids + inotropes/vasopressors  **Timing:**  Pre-operative  Post-operative | **Primary**:  Mortality  **Secondary**:  Post-operative complication rate; PA catheter complications; cost analysis | No conflict of interest declared. |
| Sinclair 1997  UK | RCT  Urgent proximal femoral fracture repair  Single centre | High | **40 randomised**  20 GDT  20 Control  **All analysed** | **Monitoring**:  OD  **Targets**:  SV optimisation with fluid boluses, FTc >0.35  **Therapy**:  Fluids only  **Timing:**  Intra-operative | **Primary**:  Hospital LoS; time to medically fit  **Secondary**:  Intra-operative haemodynamics | Project grant from the special trustees of the Middlesex Hospital. |
| Stens 2017  The Netherlands | Pragmatic RCT  Elective moderate risk general surgery  Multi-centre | Low  (Majority ASA 2) | **244 randomised**  122 GDT  122 Control  **Analysed**  81 GDT (did not receive intervention = 6, discontinued intervention = 27, excluded as high risk procedure = 8)  94 Control (did not receive intervention = 4, discontinued intervention = 18, excluded as high risk procedure = 6) | **Monitoring**:  Minimally invasive cardiac output monitor  **Targets**:  MAP >70, PPV<12%, CI >2.5  **Therapy**:  Fluids + inotropes/vasopressors  **Timing:**  Intra-operative | **Primary**:  30 day complication rate  **Secondary**:  Rate of ICU re-admission; ICU LoS; hospital LoS; hospital re-admission rate; 30 day mortality | Departmental funding. Nexfin devices supplied by Edwards Lifesciences. |
| Ueno 1998  Japan | RCT  Elective Liver surgery  Single centre | High | **34 randomised**  16 GDT  18 Control  **All analysed** | **Monitoring:**  PAC  **Targets**:  Normal: CI 2.8-4.0  Supranormal: CI >4.5, DO_2_I >600, VO_2_I >170  **Therapy:**  Fluids + inotropes/vasopressors  **Timing**:  Post-operative | **Primary**:  In-hospital mortality  **Secondary**:  Bleeding;  incidence of peritoneal infection; ARDS; hyperbilirubinaemia; liver failure. | No conflict of interest declared. |
| Valentine 1998  USA | RCT  Elective abdominal aortic reconstruction  Single centre | High | **120 randomised**  60 GDT  60 Control  **All analysed** | **Monitoring:**  PAC  **Target**:  PCWP 8-15, CI ≥2.8, SR≤1100  **Therapy**:  Fluids + inotropes/vasopressors  **Timing**:  Pre-operative | **Primary**:  Hospital LoS; all-cause mortality  **Secondary**:  Incidence of complications; PAC placement rate in control group; duration of mechanical ventilation; ICU LoS | No conflict of interest declared. |
| Van Der Linden 2010  Belgium | RCT  Elective peripheral arterial bypass grafting  Single centre | High | **57 randomised**  40 GDT  17 Control  **All analysed** | **Monitoring**:  Minimally invasive cardiac output monitor  **Target**: CI >2.5  **Therapy**:  Fluids + inotropes/vasopressors  **Timing:**  Intra-operative | **Primary**:  Mortality  **Secondary**:  Hospital LoS; incidence of post-operative complications | Departmental funding only. One author has received speaker’s fees from Abbott Laboratories. |
| Venn 2002  UK | RCT  Proximal femoral fracture repair  Single centre | High  (ASA 3 or 4) | **90 randomised**  30 GDT  60 Control  **All analysed** | **Monitoring**:  OD  **Target**:  SV optimisation with FB, FTc >400ms  **Therapy:**  Fluids only  **Timing**:  Intra-operative | **Primary**:  Time to be medically fit for discharge; hospital LoS; post-operative morbidity  **Secondary**:  Incidence of severe hypotension; differences in CVP measurements. | No conflict of interest declared. |
| Wakeling 2005  UK | RCT  Elective major large bowel surgery  Single centre | Low  (ASA 1 or 2) | **134 randomised**  67 GDT  67 Control  **Analysed**  64 GDT (did not receive intervention = 3)  64 Control (did not receive intervention = 3) | **Monitoring:**  OD  **Target**:  SV optimisation with fluid boluses  **Therapy**:  Fluids only  **Timing**:  Intra-operative | **Primary**:  Hospital LoS  **Secondary**:  Time taken to normal diet post-operatively | This study was funded by the NHS Executive South East Research and Development grant SEO252. |
| Weinberg 2017  Australia | RCT  Elective pancreaticoduodenectomy  Multi-centre | High  (Majority ASA ≥3) | **52 randomised**  26 GDT  26 Control  **All analysed** | **Monitoring**:  Minimally invasive cardiac output monitor  **Target**:  SVV <20%, MAP within 20% baseline, CI >2.0  **Therapy**:  Fluids + inotropes/vasopressors  **Timing**:  Intra-operative | **Primary**:  Hospital LoS  **Secondary**:  Fluid administration perioperatively; use of vasoactive medications; incidence of complications | No conflict of interest declared. |
| Weinberg 2019  Australia | RCT  Elective major liver resection  Mulit-centre | High  (Majority ASA 3) | **50 randomised**  25 GDT  25 Control  **Analysed**  24 GDT (flotrac not available = 1)  24 control (flotrac not available = 1) | **Monitoring**:  Minimally invasive cardiac output monitor  **Target**:  SVV (SV optimisation), CI >2.2L/min  **Therapy:**  Fluids + inotropes/vasopressors  **Timing**:  Intra-operative | **Primary**:  Hospital LOS  **Secondary**:  Number of complications per patient; number of participants with at least 1 complication; volume of intra-operative fluid; intra-operative fluid balances; use of intra-operative vasoactives. | Authors sponsored by Edwards Lifesciences  Study supported by Department of Anaesthesia Research |
| Wilson 1999  UK | RCT  Elective mixed major surgery  Single centre | High risk  (based on surgical and medical criteria) | **138 randomised**  92 GDT  46 Control  **All analysed** | **Monitoring:**  PAC  **Target**:  DO_2_I >600,  **Therapy**:  Fluids + inotropes/vasopressors  **Timing**:  Pre-operative  Intra-operative  Post-operative | **Primary**:  In-hospital morbidity and mortality  **Secondary**:  Hospital LoS  ICU/HDU LoS  Haemodynamic measurements | Grant of £45 000 from the National Hospital Lotteries Fund. |
| Wu 2017  China | RCT  Elective surgery for supratentorial neoplasms  Single centre | Low  (ASA 1 or 2) | **66 randomised**  33 GDT  33 Control  **Analysed**  33 GDT  30 Control (did not receive allocated intervention = 2, underwent repeat operation = 1) | **Monitoring**:  Minimally invasive cardiac output monitor  **Target**:  SVV<12%, CI >2.5,  **Therapy**:  Fluids + inotropes/vasopressors  **Timing:**  Intra-operative | **Primary**:  Volume of fluid administered  **Secondary**:  Biochemical measurements; post-operative complications | No conflict of interest declared. |
| Xu 2017  China | RCT  Elective thoracic lobectomy  Single centre | Low  (ASA 1 or 2) | **172 randomised**  86 GDT  86 Control  **Analysed**  84 GDT (failed to reach predefined goals = 2)  84 Control (lost to follow up = 1, severe arrhythmia = 1) | **Monitoring**:  Minimally invasive cardiac output monitor  **Target**:  SVV ≤13%, CI ≥2.5, MAP >65  **Therapy**:  Fluids + inotropes/vasopressors  **Timing**:  Intra-operative | **Primary**:  PaO_2_/FiO_2_ ratio  **Secondary**:  Other pulmonary variables and pneumodynamics; inflammatory response post-operatively;  incidence of post-operative pulmonary complications | This research was supported by the Nation Natural Science Foundation of China, the Anhui Provincial Natural Science Foundation, the Anhui Provincial Application Research. |
| Yin 2018  China | RCT  Elective laparoscopic gastrointestinal surgery  Single centre | High  (Majority ASA 3) | **50 randomised**  25 GDT  25 Control  **Analysed**  22 GDT (3 failed to complete study)  23 Control (2 failed to complete study) | **Monitoring**:  Minimally invasive cardiac output monitor  **Target**:  CI 2.5-4, SVV <13%  **Therapy**:  Fluids + inotropes/vasopressors  **Timing**:  Intra-operative | **Primary**:  Moderate or severe post-operative complications within 30 days  **Secondary**:  Return of GI function; hospital LoS | No conflict of interest declared. |
| Zakhaleva 2013  USA | RCT  Elective bowel resection with primary anastomosis  Single centre | High  (Majority ASA 3) | **91 randomised**  42 GDT  49 Control  **Analysed**  32 GDT (consent withdrawal = 1, procedure failure = 9)  42 Control (consent withdrawal = 2, no surgery = 5) | **Monitoring:**  OD  **Target**:  FTc >350, SV optimisation with fluid boluses  **Therapy**:  Fluids only  **Timing**:  Intra-operative | **Primary**:  Post-operative complication rate  **Secondary**:  Operating time (min) from first skin incision to application of the dressing; estimated blood loss (ml); intra-operative fluid (l) & post-operative fluid administered; day of out of bed; day of the first passage of flatus; removal of the epidural catheter; resumption of diet; hospital LoS; 30-day mortality | The study was supported by an unrestricted educational grant from Deltex Medical. |
| Zeng 2014  China | RCT  Elective elderly patients with hypertension who received radical gastric cancer surgery  Single centre | Low  (Majority ASA 2) | **60 randomised**  30 GDT  30 Control  **All analysed** | **Monitoring:**  Minimally invasive cardiac output monitor  **Target**:  SVV 8-13%  **Therapy**:  Fluids only  **Timing**:  Intra-operative | **Primary**:  Haemodynamic parameters and need for vasoactive drugs  **Secondary**:  Post-operative complications | The study was supported by the Joint Research Fund of Fujian Medical University |
| Zhang 2013  China | RCT  Elective thoracoscopic lobectomy  Single-centre | Low  (ASA 1-2) | **80 randomised**  30 GDT  30 Control  **60 analysed** – unclear where difference encountered | **Monitoring**:  Minimally invasive cardiac ouput monitor  **Targets**:  SVV 9-11%, CI >2.5  **Therapy**:  Fluids + inotropes/vasopressors  **Timing:**  Intra-operative | **Primary**:  PaO_2_/FiO_2_ ratio  **Secondary**:  Post-operative outcomes; hospital LoS; fluids administered; urine output; incidence of PONV |  |

Studies recruiting cardiac surgery patients, critical care patients requiring surgery, high risk surgery, or more than 50% of patients with ASA class 3 or higher were classified as having ‘high risk’ patients

LoS = length of stay

**Figure A**: review authors' judgements about each risk of bias item presented as percentages across all included studies. Green indicates no risk of bias, yellow and red represents unclear risk and high risk respectively.

**Search strategy**

**Embase**

1. exp surgery/
2. perioperative period/
3. intraoperative period/ or perioperative care/
4. preoperative care/
5. exp surgical technique/
6. (Surg* or operati* or Peri?operati* or intra?operati* or post?operati* or resect* or repair or transplant or surgical procedures).ab,ti.
7. 1 or 2 or 3 or 4 or 5 or 6
8. heart output/
9. heart stroke volume/
10. ((cardiac adj3 (output or index)) or (stroke adj3 (output or index)) or (oxygen adj3 (delivery or consumption or saturation or extraction)) or lactat* or CVP or SVO2 or VO2 or DO2 or tonometry or venous oxygen saturation).ab,ti.
11. 8 or 9 or 10
12. swan ganz catheter/
13. exp doppler echocardiography/
14. hemodynamics/
15. (fluid therapy/ or exp rehydration/).ab,ti.
16. (goal?directed or h?emodynamic or optimisat* or optimizat* or GDFT or GDT or GDHT or fluid therapy or fluid admi* or vasoactive or pulmonary artery catheter or PAFC or Swan Ganz or doppler or PICCO* LIDCO* or lithium dilution or thermodilution or flotrac or vigileo or echocardio*).ab,ti.
17. 12 or 13 or 14 or 15 or 16
18. randomized controlled trial/
19. controlled clinical study/
20. random$.ti,ab.
21. randomization/
22. 18 or 19 or 20 or 21
23. 7 and 11 and 17 and 22

**OVID Medline**

1. exp specialties, surgical/
2. perioperative care/ or intraoperative care/ or perioperative nursing/ or postoperative care.mp.
3. exp surgical procedures, operative/
4. (Surg* or operati* or Peri?operati* or intra?operati* or post?operati* or surgical procedures).mp.
5. 1 or 2 or 3 or 4
6. cardiac output/ or stroke volume/
7. ((cardiac adj3 (output or index)) or (stroke adj3 (output or index)) or (oxygen adj3 (delivery or consumption or saturation or extraction)) or lactat* or CVP or SVO2 or VO2 or DO2 or tonometry or venous oxygen saturation).mp.
8. 6 or 7
9. catheterization, Swan-Ganz/
10. exp echocardiography, doppler/
11. hemodynamics/
12. exp fluid therapy/
13. (goal?directed or h?emodynamic or optimisat* or optimizat* or GDFT or GDT or GDHT or fluid therapy or fluid admi* or vasoactive or pulmonary artery catheter or PAFC or Swan Ganz or doppler or PICCO* LIDCO* or lithium dilution or thermodilution or flotrac or vigileo or echocardio*).mp.
14. 9 or 10 or 11 or 12 or 13
15. (randomized controlled trial or controlled clinical trial or randomized or randomised or trial or groups).mp.
16. 5 and 8 and 14 and 15 (7712)

**Central**

#1 MeSH descriptor: [Specialties, Surgical] explode all trees

#2 (surg* or operati* or Peri?operati* or intra?operati* or post?operati* or surgical procedures)

#3 (cardiac near (output or index) or stroke near (output or index) or oxygen near (delivery or consumption or saturation or extraction) or lactate or CVP or SVO2 or VO2 or DO2 or tonometry or venous oxygen saturation)

#4 MeSH descriptor: [Catheterization, Swan-Ganz] explode all trees

#5 MeSH descriptor: [Echocardiography, Doppler, Pulsed] explode all trees

#6 (goal?directed or h?emodynamic or optimisat* or optimizat* of GDFT or GDT or GDHT or fluid therapy or fluid admi* or vasoactive or pulmonary artery catheter or PAFC or Swan Ganz or doppler or PICCO* or LIDCO* or lithium dilution or thermodilution or flotrac or vigileo or echocardio*)

#7 #1 or #2

#8 #4 or #5 or #6

#9 #7 and #3 and #8
